# TRPM7 contributes to progressive nephropathy

**DOI:** 10.1038/s41598-020-59355-y

**Published:** 2020-02-11

**Authors:** Sayuri Suzuki, Reinhold Penner, Andrea Fleig

**Affiliations:** 1 Center for Biomedical Research, The Queen’s Medical Center, 1301 Punchbowl St., Honolulu, HI 96813 USA; 2 University of Hawaii Cancer Center, University of Hawaii, 651 Ilalo St., Honolulu, HI 96813 USA; 3 John A. Burns School of Medicine, University of Hawaii, 651 Ilalo St., Honolulu, HI 96813 USA

**Keywords:** Cell biology, Drug discovery, Molecular biology, Diseases, Nephrology

## Abstract

TRPM7 belongs to the Transient Receptor Potential Melastatin family of ion channels and is a divalent cation-conducting ion channel fused with a functional kinase. TRPM7 plays a key role in a variety of diseases, including neuronal death in ischemia, cancer, cardiac atrial fibrillation, malaria invasion. TRPM7 is aberrantly over-expressed in lung, liver and heart fibrosis. It is also overexpressed after renal ischemia-reperfusion, an event that induces kidney injury and fibrosis. However, the role of TRPM7 in kidney fibrosis is unclear. Using the unilateral ureteral obstruction (UUO) mouse model, we examined whether TRPM7 contributes to progressive renal damage and fibrosis. We find that TRPM7 expression increases in UUO kidneys. Systemic application of NS8593, a known TRPM7 inhibitor, prevents kidney atrophy in UUO kidneys, retains tubular formation, and reduces TRPM7 expression to normal levels. Cell proliferation of both tubular epithelial cells and interstitial cells is reduced by NS8593 treatment in UUO kidneys, as are TGF-β1/Smad signaling events. We conclude that TRPM7 is upregulated during inflammatory renal damage and propose that pharmacological intervention targeting TRPM7 may prove protective in progressive kidney fibrosis.

## Introduction

TRPM7 belongs to the Transient Receptor Potential (TRP) Melastatin family of ion channels, but is unique in that its ion channel domain is fused with a functional serine/threonine kinase^1^. TRPM7 was the first ion channel shown to conduct a range of divalent cations^2,3^, including physiologically essential divalent cations such as Ca^2+^, Mg^2+^, Zn^2+^, Ni^2+^, Mn^2+^, and Co^2+^. Ample evidence supports TRPM7 to be critical in regulating Mg^2+^ homeostasis^1^ and play a key role in a variety of diseases, including neuronal death in ischemia^4^, renal ischemia^5^, and cardiac atrial fibrillation^6^. TRPM7 is expressed ubiquitously in mammals, and is key in driving cell growth and proliferation, linking the protein to hyperproliferative diseases such cancer^1,7,8^ and pulmonary, hepatic, and cardiac fibrosis^6,9–12^. Tissue fibrosis refers to a number of conditions that cause interstitial organ damage followed by inflammation and maladaptive wound healing eventually leading to fibrosis and chronic disease in the organ affected. TRPM7 overexpression leads to fibrosis through the TGF-β signaling pathway^9–11^. Recent *in vitro* data show that TRPM7 plays a pivotal role in progressive inflammatory and fibrotic disease by promoting excessive extracellular matrix (ECM) deposition^9^. It regulates proliferation and differentiation of fibroblasts^11^ and proliferation and polarization of macrophages^13^ as well as their Ca^2+^-dependent activation^14^. A recent study supports this hypothesis, as genetic suppression of TRPM7 in a kidney transplantation mouse model alleviated progressive inflammatory kidney fibrosis^15^.

In the kidney, cell proliferation is a central response to injury that culminates in the development of fibrosis and renal failure. Unilateral ureteral obstruction (UUO) is a widely used mouse model of kidney disease associated with progressive tubulointerstitial injury^16–18^. It has been used to identify many of the molecular and cellular events that occur in progressive kidney fibrosis. After obstruction of the kidney, increased hydrostatic pressure and decreased renal blood flow and glomerular filtration stimulate the infiltration of monocytes and cytokine release to promote macrophage invasion into the renal tubular interstitium. Activated macrophages produce cytokines, including tumor necrosis factor-α (TNF-α) and transforming growth factor-β1 (TGF-β1), to increase cell proliferation of renal tubular epithelial cells and activation of tubular interstitial myofibroblasts (transformed fibroblasts expressing α-SMA)^18–20^. The myofibroblasts produce excessive ECM, including deposits of collagen, fibronectin and vimentin. The TGF-β1/Smad signaling pathway stimulates tubular epithelial cells to trans-differentiate into mesenchymal cells^18,19^, which infiltrate the renal tubular interstitium through the broken tubular basement membrane. Trans-differentiated mesenchymal cells further differentiate into ECM-producing myofibroblasts, thus undergoing hyperproliferation and resulting in irreversible progression of renal interstitial fibrosis^18^. Thus, cell proliferation critically contributes to progressive kidney fibrosis.

Unfortunately, there are no treatment modalities that are effective in halting the progression of most chronic kidney diseases associated with kidney fibrosis^21^. NS8593 is a small conductance potassium channel inhibitor that also significantly affects TRPM7^22,23^ and has been shown to inhibit the proliferation and polarization of human macrophages mediated by TRPM7^13^. It therefore represents a useful pharmacological tool to investigate the role of TRPM7 in the pathophysiology of kidney fibrosis and evaluating this channel as a potential therapeutic target.

## Results

### TRPM7 expression is upregulated in renal damage

**Figure 1:**
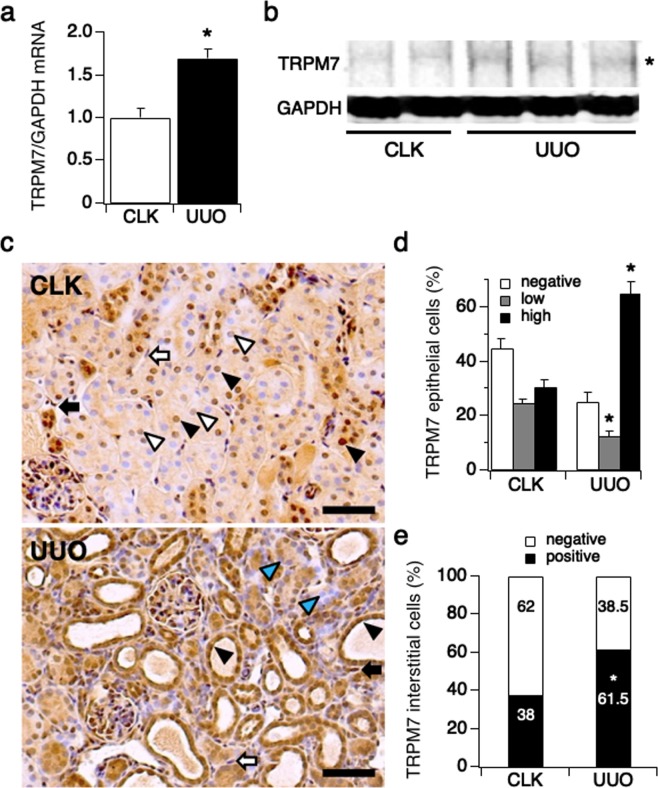
TRPM7 expression is up-regulated in renal damage. The left kidney was obstructed in C57BL/6 mice by ureter ligation (UUO mouse model, see Methods) inducing progressive kidney damage^17,28^ (n = 5). After 7 days, obstructed (UUO kidneys) and non-obstructed contralateral kidneys (CLK kidneys) were collected from sacrificed mice. (**a**) The level of TRPM7 mRNA expression was assessed in tissue from cortical kidney using qRT-PCR. (*p < 0.001). (**b**) The level of TRPM7 protein expression was examined in cortical kidney tissue using western blotting. The full-length gel is presented in Suppl. Fig. S1c. (**c**) Upper panel: Representative picture of immunostaining against TRPM7 (magnification x200) in CLK kidneys, where white triangular arrows indicate low expression- and black triangular arrows high expression levels of TRPM7 in renal tubular epithelial cells. White arrows point to TRPM7-negative (TRPM7 staining not detectable) and black arrows point to TRPM7-positive renal interstitial cells. Lower panel: Representative picture of TRPM7 immunostaining in UUO kidneys, where TRPM7-high expression epithelial cells preferentially are localized in dilated renal tubules (black triangular arrow) and TRPM7-negative epithelial cells are localized in non-dilated tubules (blue triangular arrow) at day 7 after surgery. Scale bars represent 100 μm. (**d**) The percentage of TRPM7-high expression (black bar), TRPM7-low expression (gray bar) and non-detectable TRPM7 staining (white bar) in tubular epithelial cells assessed in a total of 10 non-overlapping fields. The average tubular epithelial cell number per field was 513 cells in CLK kidneys and 589 cells in UUO kidneys. *p < 0.001 vs. CLK kidneys. (**e**) The percentage of TRPM7-positive (black bar) or TRPM7-negative (non-detectable, white bar) interstitial cells assessed at same fields in (**d**). The average interstitial cell number per field was 121 cells in CLK kidneys and 444 cells in UUO kidneys. *p < 0.001 vs. CLK kidneys.

The role of TRPM7 in kidney damage is not well understood. We took advantage of a well-established mouse model mimicking progressive tubulointerstitial injury induced by unilateral ureteral obstruction (UUO, see methods)^16,17,20^. We first assessed the level of TRPM7 mRNA expression in obstructed kidneys (UUO) and compared this to non-obstructed contralateral control kidneys (CLK) isolated from UUO mice sacrificed at day 7 (Fig. 1a; n = 5). We found that TRPM7 mRNA was significantly upregulated in obstructed (UUO) kidneys.

We next tested for TRPM7 protein expression in CLK and UUO kidneys using western blot (WB) and immunohistochemistry. In preparation for this, we first confirmed that the commercially available TRPM7 antibody used (see methods) was able to detect TRPM7 protein, for both species, human and mouse. To this end, we performed WB on cell lysate derived from non-induced (0 hrs) or tetracycline (Tet)-induced HEK293-TReX cells overexpressing human TRPM7 (hTRPM7; 15 hrs induction time) in the presence or absence of the corresponding TRPM7 antibody blocking peptide (Suppl. Fig. S1a; see methods for peptide information). We found that the TRPM7 antibody detected hTRPM7 (Suppl. Fig. S1a, lane 2), and this detection was blocked by pre-incubation with the TRPM7 blocking peptide (Suppl. Fig. S1a, lane 4). To demonstrate that the cell lysate derived from the HEK293-TReX cells overexpressing hTRPM7 was loaded appropriately on lane 4, we re-probed the same membrane with TRPM7 antibody (Suppl. Fig. S1a; re-probe panel, lanes 3 and 4). Indeed, TRPM7 protein was detected by the TRPM7 antibody in the absence of the blocking peptide. Similarly, and in preparation of the immunohistochemistry on mouse kidney tissue, the specificity of the TRPM7 antibody was confirmed by the loss of immunoreactivity of TRPM7-positive cells in mouse kidney tissue when using the blocking peptide for the TRPM7 antibody (Suppl. Fig. S1b).

Next, we evaluated TRPM7 protein levels by WB using above TRPM7 antibody. In analogy to the mRNA data (Fig. 1a), we found that TRPM7 was significantly up-regulated in UUO compared to CLK kidneys in mouse (Fig. 1b, Suppl. Fig. S1c,d).

When evaluating individual renal tubular epithelial cells for TRPM7 staining by immunohistochemistry, our analysis of 10 fields from each kidney revealed 3 patterns of TRPM7 staining in tubular epithelial cells for both CLK and UUO kidneys (Fig. 1c): a strong positive staining (TRPM7 high expression, black arrow head in Fig. 1c upper panel), a slight positive staining (TRPM7 low expression, white arrow head in Fig. 1c upper panel), and TRPM7 negative staining (Fig. 1c, cells appearing blue). Quantification of these data showed that 24% of CLK epithelial cells had low expression of TRPM7 (Fig. 1d; gray bar) and 31% of cells showed strongly expressed TRPM7 (Fig. 1d; black bar). The remaining 45% of cells did not show detectable TRPM7 staining (Fig. 1d; open bar). In contrast, UUO kidneys displayed a dramatic increase in strongly TRPM7-expressing epithelial cells to 65% of the analyzed cell population and reduced proportions of lightly expressing TRPM7 cells to 13% and TRPM7 negative cells to 25% of the cell population (Fig. 1d).

Interestingly, high TRPM7 expression was predominantly seen in epithelial cells located within dilated renal tubules (Fig. 1c; lower panel, black arrows), and TRPM7-negative cells were largely found in non-dilated renal tubules in UUO kidneys (Fig. 1c; lower panel, blue arrows). In addition, the number of TRPM7 protein-positive cells of another important cell type involved in kidney fibrosis, tubular interstitial cells, also significantly increased in UUO kidneys compared to CLK (62% in UUO vs. 38% in CLK kidneys, Fig. 1e; black bars). We conclude that overexpression of TRPM7 is an indicator of progressive kidney damage in UUO kidneys of mouse.

### NS8593 suppresses manganese influx in epithelial and fibroblast rat kidney cell models

**Figure 2:**
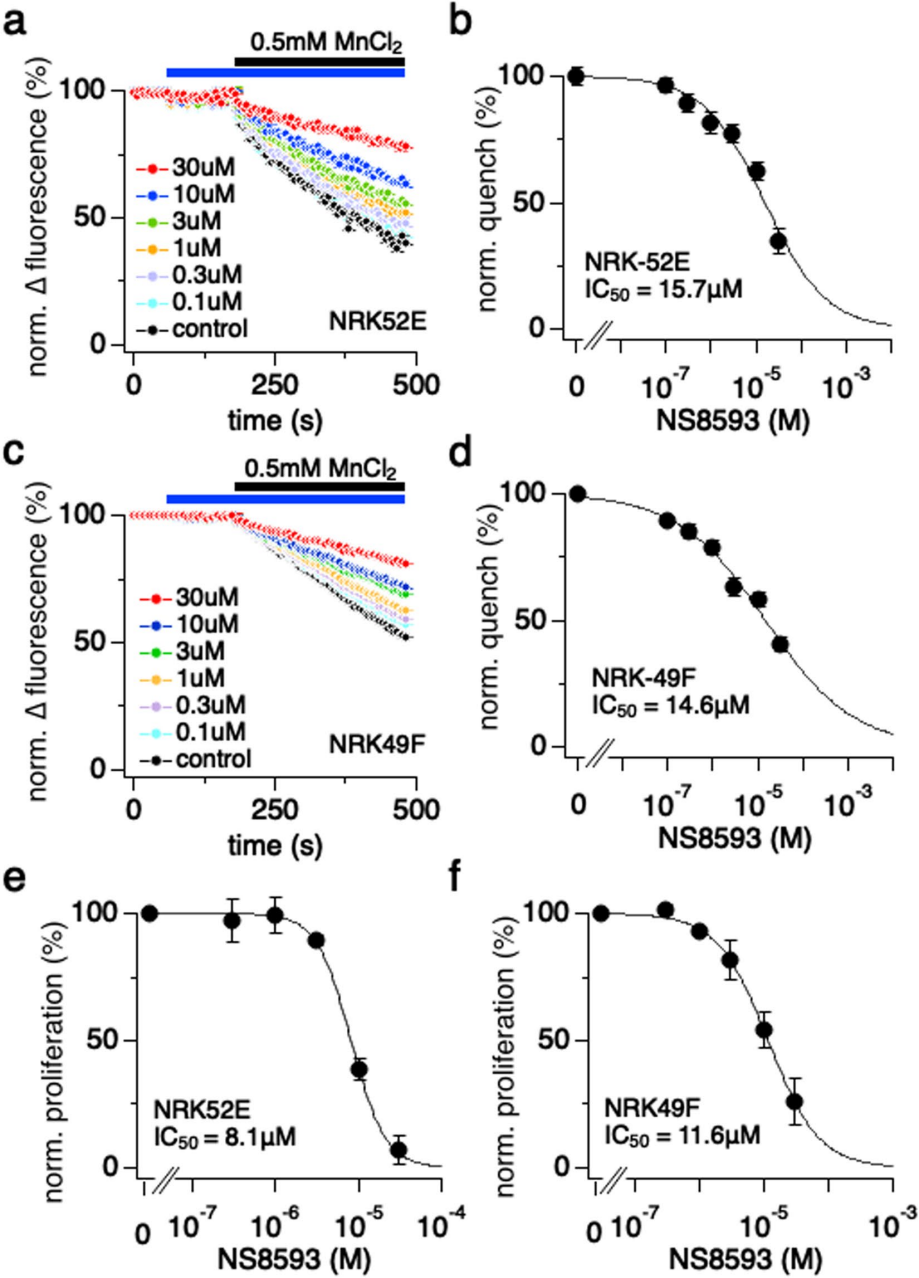
NS8593 suppresses Mn^2+^ influx and cell proliferation in epithelial and fibroblast kidney cells. (**a**) NRK-52E cells were investigated for the inhibitory efficacy of NS8593 in a TRPM7-specific bioassay^24,25^ using Fura-2AM Mn^2+^ quench assessed with a fluorescent kinetic plate reader in 96-well format. The excitation wavelength was 360 nm. Control (0 μM), 0.1, 0.3, 1, 3, 10, 30 μM of NS8593 was added at 61 s for pre-incubation (blue bar). In a second application, the final concentration of 0.5 mM MnCl_2_ was added to each well at 181 s (black bar) (n = 4). (**b**) The graph shows the dose-response behavior of Mn^2+^ quench in response to increasing concentrations of NS8593. The percent quench of Fura-2AM fluorescence was assessed at 480 s into the experiment, averaged, plotted versus time and approximated with a dose-response fit. (**c**) Same experimental outline as in (**a**), assessed in the NRK-49F cell line (n = 4). (**d**) The graph shows the dose-response behavior of Mn^2+^ quench as in (**b**), assessed in the NRK-49F cell line. (**e**) The inhibitory effect of NS8593 on cell proliferation was examined in NRK-52E cells using the MTT assay (see Methods). Cells were incubated with increasing concentrations of NS8593 in a 96-well plate for 2 days, after which percent cell viability was evaluated, averaged, plotted and approximated with a dose-response fit (n = 3). (**f**) Same MTT experiment as in (**e**) but with NRK-49F cells (n = 3). Here cells were incubated with increasing concentrations of NS8593 for 3 days before the MTT analysis. The plate medium was exchanged every 24 hours with fresh medium supplemented with appropriate NS8593 concentrations.

We next asked the question whether pharmacological suppression of TRPM7 channel activity would have protective effects in the UUO animal model. To this end, we first assessed the ability of NS8593 – a small conductance potassium channel inhibitor that very efficiently suppresses TRPM7 currents as well^13,22,23^ – to suppress intracellular fluorescence quenching of Fura-2 by manganese (Mn^2+^) ions permeating through TRPM7^24,25^ in epithelial kidney NRK-52E and fibroblast NRK-49F model cells. We performed a dose-response analysis for NS8593 on intracellular Fura-2 quench induced by Mn^2+^ binding in both cell lines using a fluorescent 96-well kinetic plate reader^24,25^ (Fig. 2a,b). Cells were exposed to various concentrations of NS8593 for 3 minutes (Fig. 2a,b; blue bar) before addition of 0.5 mM MnCl_2_ to the external bath solution (Fig. 2a,b; black bar). The maximum value was measured in the absence of any inhibitor (Fig. 2a,b; black circles) and Mn^2+^ quench was assessed measuring the fluorescence levels obtained at the end of the experiment. Increasing concentrations of NS8593 reduced Mn^2+^ quench in a dose-dependent manner with an IC_50_ of 16 ± 3 µM in NRK-52E cells (n = 4) and NRK-49F with an IC_50_ of 15 µM ± 2 µM (Fig. 2c,d).

### NS8593 reduces cell proliferation in kidney cell models

It is well known that TRPM7 is mandatory for cell proliferation in many types of tissue and accordingly TRPM7 inhibitors have been shown to interfere with this process^25,26^. We therefore performed MTT assays to investigate the effect of NS8593 on NRK-52E and NRK-49F cell proliferation to complement our Mn^2+^ quench bioassay. Figure 2e,f show that NS8593 treatment reduced cell proliferation in a dose-dependent manner similar to the results of our Mn^2+^ quench bioassays with IC_50_ values of 8 µM in NRK-52E (Fig. 2e), and 12 μM in NRK-49F cells (Fig. 2f). To investigate whether the inhibitory effect of NS8593 on cell proliferation is mediated by TRPM7 inhibition, we performed siRNA-mediated TRPM7 knockdown in NRK52-E and NRK-49F cells and then used MTT assays to assess cell proliferation at different NS8593 concentrations. (Suppl. Fig. S2a). siRNA caused a 68% decrease of TRPM7 mRNA in NRK-49F and somewhat smaller reduction of 32% in NRK-52E (black bars). If NS8593 were acting on an unrelated mechanism, we would expect additive or even supra-additive effects to siRNA. However, the inhibition of cell proliferation by NS8593 merely shifted the potency of NS8593 inhibition in NRK-49F (16 µM to 43 µM) and to a lesser extent in NRK-52E (7 to 10 µM) with no significant additivity to TRPM7-mediated inhibition (see Suppl. Fig. S2). We cannot entirely rule out some potential off-target effects at higher concentrations, which could then reflect the combination of both less-specific effects and a block of residual TRPM7 channels spared by the siRNA-mediated knockdown. This could also explain why the lower efficacy in siRNA knockdown in NRK-52E cells was paralleled by a smaller shift in IC_50_. Together, these data suggest that the inhibition of cell proliferation is at least partially mediated by NS8593-mediated inhibition of TRPM7.

### NS8593 directly blocks TRPM7 currents

**Figure 3:**
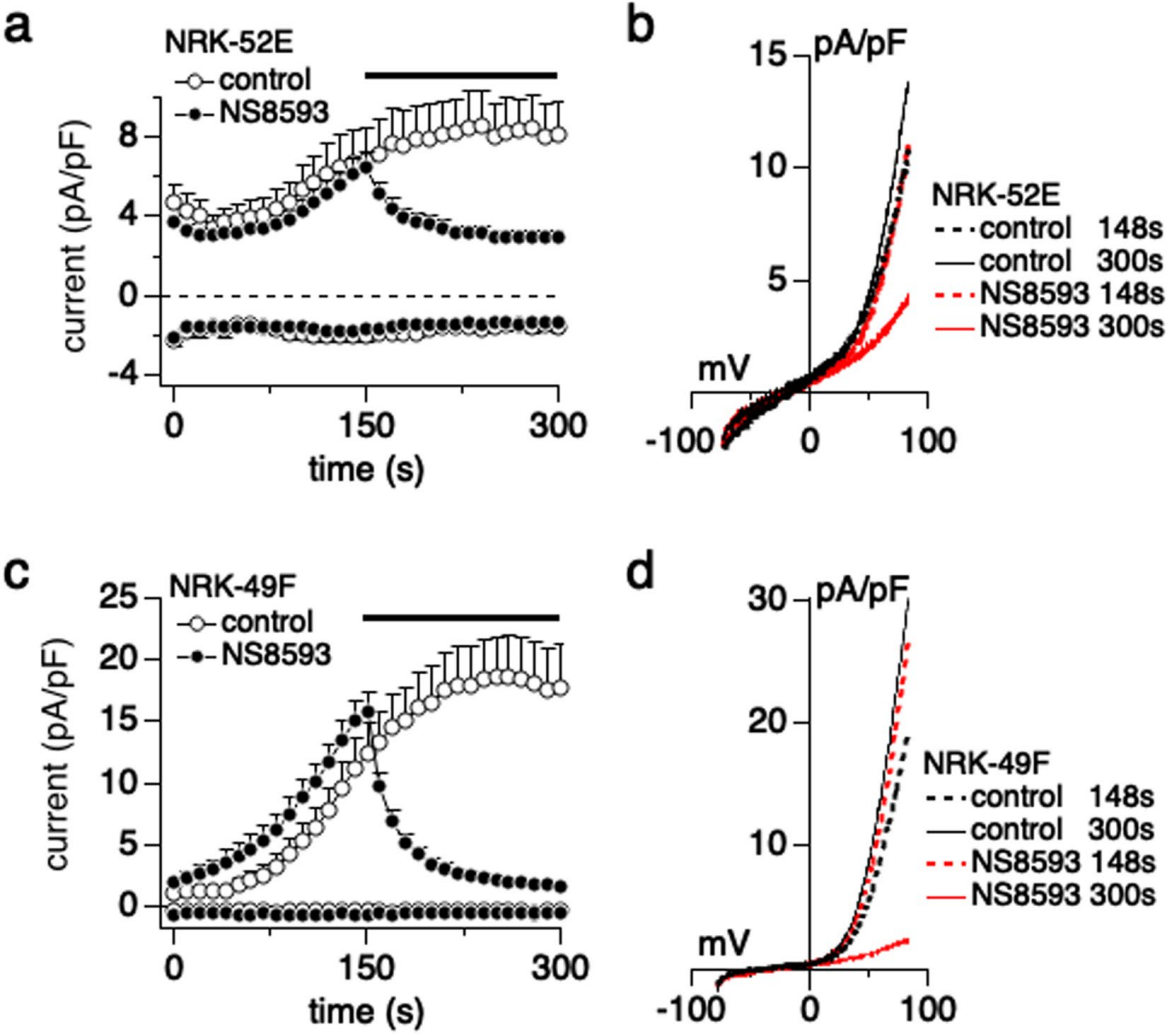
NS8593 blocks TRPM7 currents in kidney cells. (**a**) Inhibitory effects of NS8593 on TRPM7 current in NRK-52E cells was investigated using whole-cell patch-clamp recordings. Average TRPM7-mediated outward currents at +80 mV and inward currents at −80 mV extracted from ramp currents delivered at 0.5 Hz and plotted as a function of time. 10 μM NS8593 (black circle, n = 15) was applied from 150 s (black bar). 0 μM NS8593 contained DMSO vehicle and was used as control (white circle, n = 8). (**b**) Average I/V curve of TRPM7 currents extracted before (148 s, dot lines) and after (300 s, solid lines) NS8593 application. Black lines represent control cells and red lines are NS8593 treatment cells. (**c**) Same experimental protocol as in (**a**), but recorded in NRK-49F cells. Black circles represent 10 μM NS8593 application (n = 11) and white circles represent vehicle control (n = 6). (**d**) Average I/V curve of TRPM7 on NRK-49F as in (**b**).

While NS8593 has previously been demonstrated to inhibit TRPM7 channel activity, we verified the direct inhibitory efficacy of NS8593 on TRPM7 currents in kidney cells by performing patch-clamp experiments. 10 µM NS8593 completely blocked TRPM7 currents in NRK-52E cells (Fig. 3a black circles and Fig. 3b red lines, n = 15) compared to control (Fig. 3a white circles and Fig. 3b black lines, n = 8). NRK-49F cells exhibited larger TRPM7 currents than NRK-52E cells (Fig. 3c,d), but 10 µM NS8593 also completely suppressed TRPM7 currents in NRK-49F cells (Fig. 3c black circles and Fig. 3d red line, n = 11), whereas DMSO vehicle control application had no effect (Fig. 3d white circles and Fig. 3d black lines, n = 6).

### NS8593 attenuates kidney atrophy in the UUO mouse model

**Figure 4:**
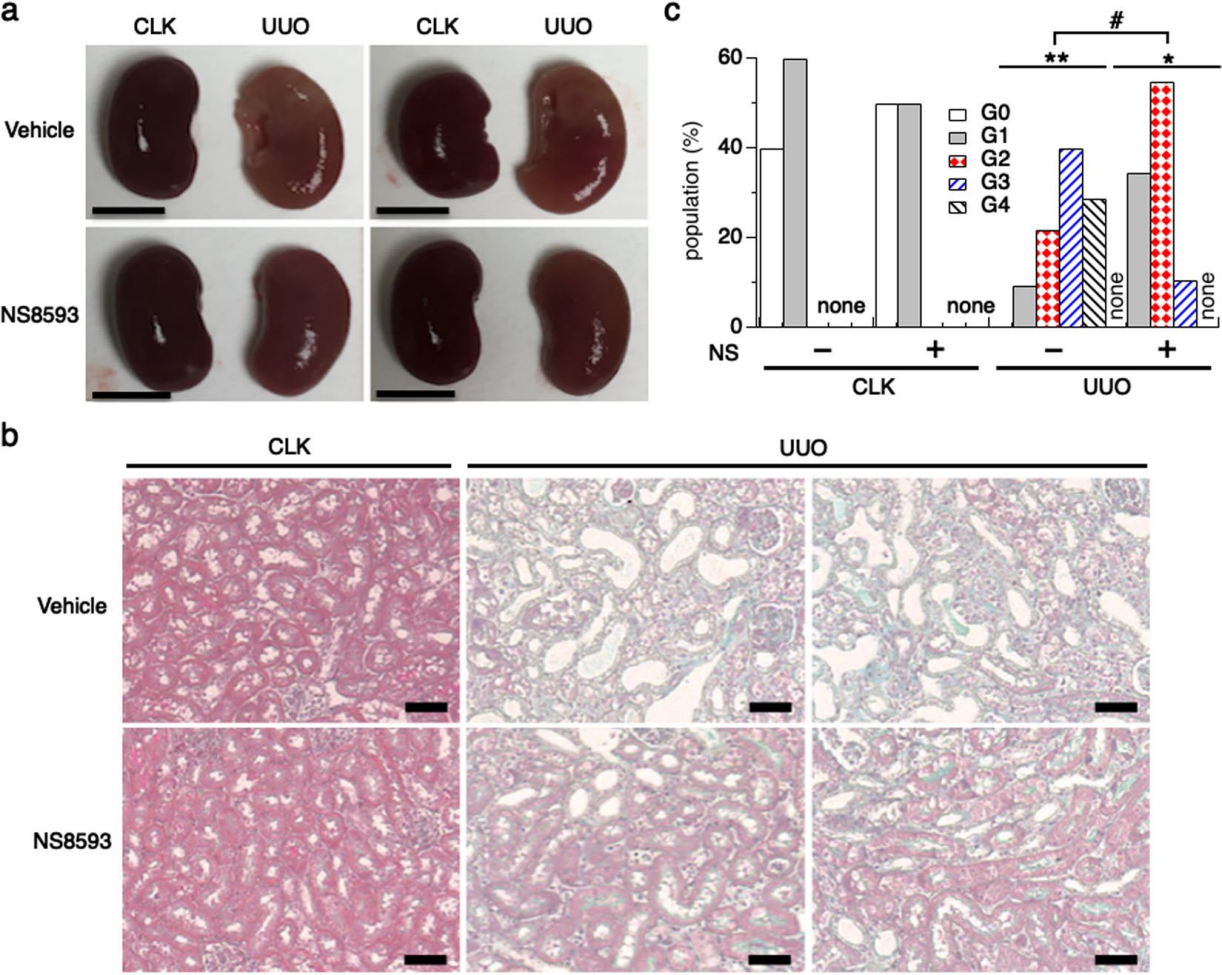
NS8593 attenuates kidney atrophy in the UUO mouse model. (**a**) Pictures of representative CLK and UUO kidneys isolated from UUO mice at day 7 after ureteral obstruction surgery. The non-treatment control group was injected with vehicle daily (n = 5, upper panels). NS8593 (5 mg/kg) was injected intraperitoneally daily from post-surgery day 0 to day 6 (n = 4, lower panel). Scale bars depict 5 mm. (**b**) Representative pictures of Masson’s trichrome staining taken from one CLK and two representative UUO kidney sections (magnification x200, see Methods). Collagen fibers, an indicator of degree of fibrosis, appear in green. The cytoplasm appears in light red. UUO kidneys in the NS8593 treatment group (lower right panels) have substantially reduced manifestation of green compared to control (upper right panels). Scale bars, 100 μm. (**c**) The intensities in (**b**) were graded semi-quantitatively^17,28^ as follows: G0 (grade 0, 0%, white bars); G1 (grade 1, ≤20% in interstitial area colored green, grey bars); G2 (grade 2, 21–50%, red dot bars); G3 (grade 3, 51–80%, blue line bars) and G4 (grade 4, ≥81%, black line bars). Average grading of each mouse was used for statistical analysis. *p < 0.005; CLK vs. UUO kidney with NS8593 treatment, **p < 0.001; CLK vs. UUO kidney with non-treatment, ^#^p < 0.005 comparing UUO kidneys in non-treatment vs. NS8593 treatment groups.

Since NS8593 showed inhibitory efficacy in our cellular TRPM7 influx and MTT cell proliferation bioassays, we next assessed the effect of NS8593 for anti-renal damage and anti-fibrosis targeting TRPM7 in UUO mice (see Methods). In the non-treatment control group injected with vehicle, UUO kidneys were flatter and renal atrophy was evident when compared with CLK kidneys (n = 5, Fig. 4a upper panels). However, UUO kidneys were able to maintain their morphology and less kidney atrophy was observed in the treatment group injected with NS8593 (n = 4, Fig. 4a lower panels). There was no overall difference in body weight between treatment and non-treatment groups (Suppl. Fig. S3a). However, NS8593 administration slightly attenuated the weight loss seen in UUO kidneys compared to CLK kidneys (−7% vs. −10% in UUO kidneys in control) albeit not in a statistically significant way (Suppl. Fig. S3b). UUO kidneys showed strong tubular dilation and tubule loss in the non-treatment group (Fig. 4b, upper panels), whereas NS8593 reduced renal tubule loss (90 vs. 70 tubule numbers in one field, Suppl. Fig. S3c), and many non-dilated tubules remained in UUO kidneys (Fig. 4b, lower panels).

We next assessed changes in interstitial areas for symptoms of fibrosis (Fig. 4b; colored in green). Levels of fibrotic tubulointerstitial lesions were graded between G0 and G4^17,27,28^ (Fig. 4C; see Methods]. Each level represents the following: G0 = no damage (white bar); G1 = weak (yellow bar); G2 = mild (red bar); G3 = moderate (blue bar), and G4 = severe damage (black bar). This analysis revealed that CLK kidneys showed no damage (G0) or a very weak damage (G1) in both the non-treatment and NS8593 treatment groups. Non-treated UUO kidneys showed severe (G4; 29%) and moderate (G3; 40%) damage. In comparison, tubulointerstitial lesions in UUO kidneys treated with NS8593 were significantly reduced compared to UUO kidneys in the non-treatment group. Furthermore, no severe damage was observed in the treatment group, and most of the cortex area displayed only mild tissue damage (G2; 55%, Fig. 4c). Comparing the average grading, there was a significant difference between NS8593-treated and untreated UUO kidneys (Fig. 4c). Based on these findings we conclude that renal tubule atrophy and kidney damage are protected by administration of NS8593.

### NS8593 suppresses TRPM7 expression in renal damage

**Figure 5:**
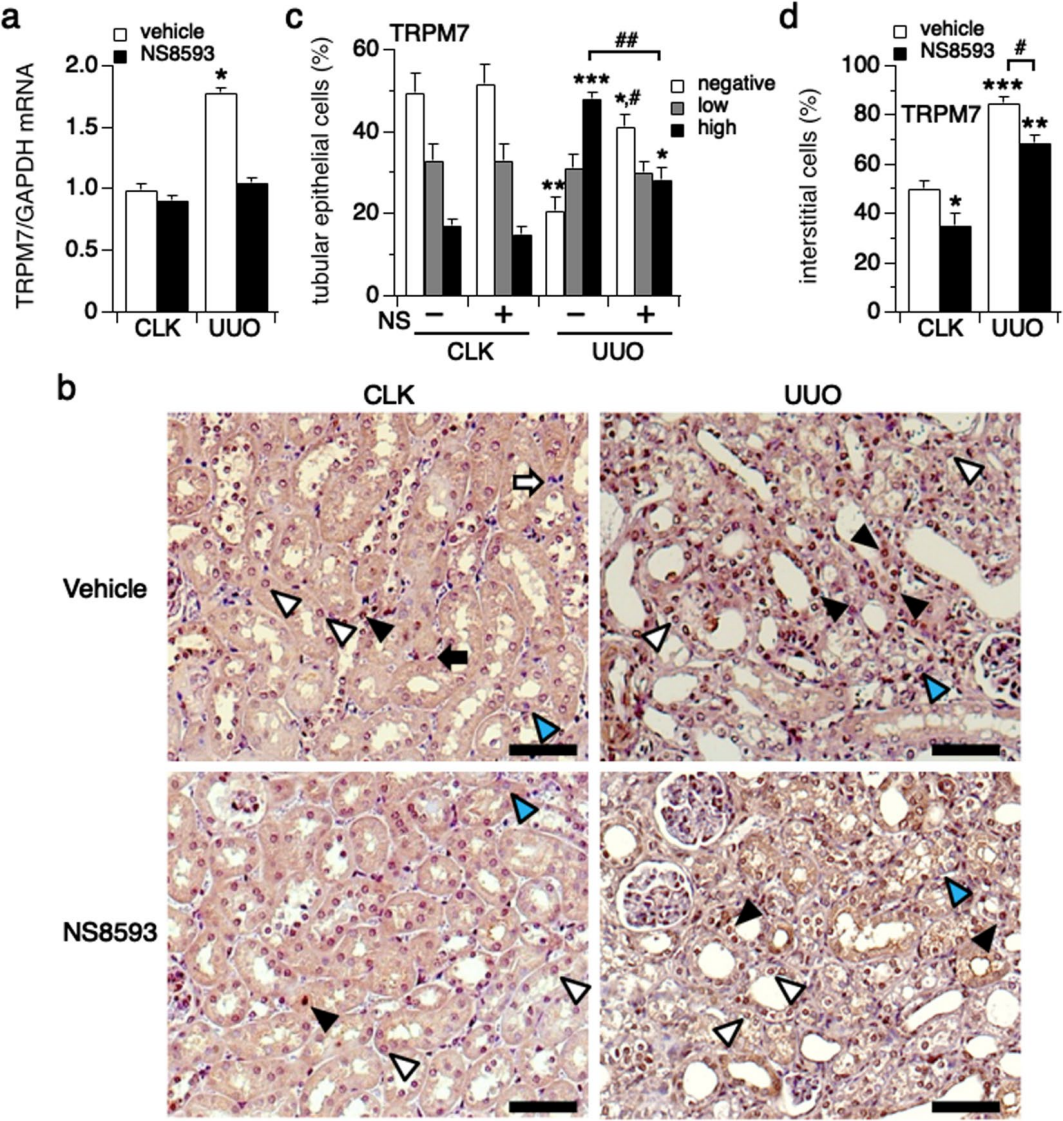
NS8593 suppresses TRPM7 expression in renal damage. (**a**) The level of TRPM7 mRNA expression was examined using UUO kidneys with (black bars) or without NS8593 treatment (white bars). *p < 0.005 compared to CLK kidneys in the non-treatment control group. (**b**) Representative slides of TRPM7 immunostaining (magnification x200) in the non-treatment (upper panels) and NS8593 treatment group (lower panels) both from CLK (left panels) and UUO kidneys (right panels). Scale bars are 100 μm. Triangular arrows indicate epithelial cells with TRPM7-high expression (black), TRPM7-low expression (white) and non-detectable (blue). Arrows point to interstitial cells that are either TRPM7-positive (black) or TRPM7-negative (white). (**c**) The graph plots the analysis of TRPM7 expression in tubular epithelial cells assessed in 10 representative and non-overlapping slides (negative, white bars; low expression, grey bars, high expression, black bars). The average tubular epithelial cell number per field was 338 cells in non-treatment CLK kidneys, 359 cells in CLK kidneys with NS8593 treatment, 418 cells in non-treatment UUO kidneys and 396 cells in UUO kidneys with NS8593 treatment. *p < 0.05 vs. CLK kidney with NS8593 treatment, **p < 0.005, ***p < 0.001 vs. CLK kidney with non-treatment, ^#^p < 0.005, ^##^p < 0.001; UUO kidneys with non-treatment vs. NS8593 treatment. (**d**) Percentage of TRPM7-positive interstitial cells assessed in non-treatment control kidneys (white bars) versus NS8593 treatment (black bars). The average interstitial cell number per field was 90 cells in non-treatment CLK kidneys, 104 cells in CLK kidneys with NS8593 treatment, 207 cells in non-treatment UUO kidneys and 142 cells in UUO kidneys with NS8593 treatment. *p < 0.05 vs. CLK kidney with non-treatment, **p < 0.005 vs. CLK kidney with NS8593 treatment, ***p < 0.001 vs. CLK kidney with non-treatment, ^#^p < 0.005 comparing UUO kidneys in non-treatment vs. NS8593 treatment groups.

We next asked whether NS8593 would have an effect on overall TRPM7 expression *in vivo* in the UUO mouse model. In analogy to our initial experiments (Fig. 1), TRPM7 mRNA was significantly upregulated in UUO kidneys compared to the contralateral kidney (CLK) (Fig. 5a; n = 4–5). However, NS8593 treatment completely prevented upregulation of TRPM7 mRNA in UUO kidneys. This inhibitory effect by NS8593 was also observed for TRPM7 protein levels (Suppl. Fig. S4), although endogenous TRPM7 in cortical kidney tissue was difficult to detect by western blot as it yielded only faint TRPM7 protein bands. The reason for this might be that cortical kidney tissue contains many cell types with relatively low TRPM7 levels. Therefore, immunostaining may be more suitable and informative for evaluation and localization of TRPM7 protein expression. Investigating TRPM7 protein levels using immunohistochemistry (Fig. 5b–d) indeed confirmed this observation. NS8593 both significantly increased the number of renal epithelial cells that did not show any detectable TRPM7 staining (20% in the non-treatment group to 37% in the treatment group; Fig. 5b,c; white bars) in UUO kidneys, while strongly reducing the number of cells with high TRPM7 expression (33% vs. 48% in the non-treatment control group, Fig. 5b,c; black bars). At the same time, no significant change was observed in the number of cells with low TRPM7 protein expression (Fig. 5c; gray bars).

When analyzing interstitial cells, TRPM7 staining was strongly increased in UUO kidneys, and this was partially reversed by administration of NS8593 (85% vs. 69%, respectively, Fig. 5b,d). At the same time, NS8593 seemed to affect TRPM7 protein levels not only in UUO but in CLK kidneys as well. We conclude that NS8593 attenuates the increase of TRPM7 expression that is caused by progressive renal damage.

### NS8593 inhibits cell proliferation in nephropathy

**Figure 6:**
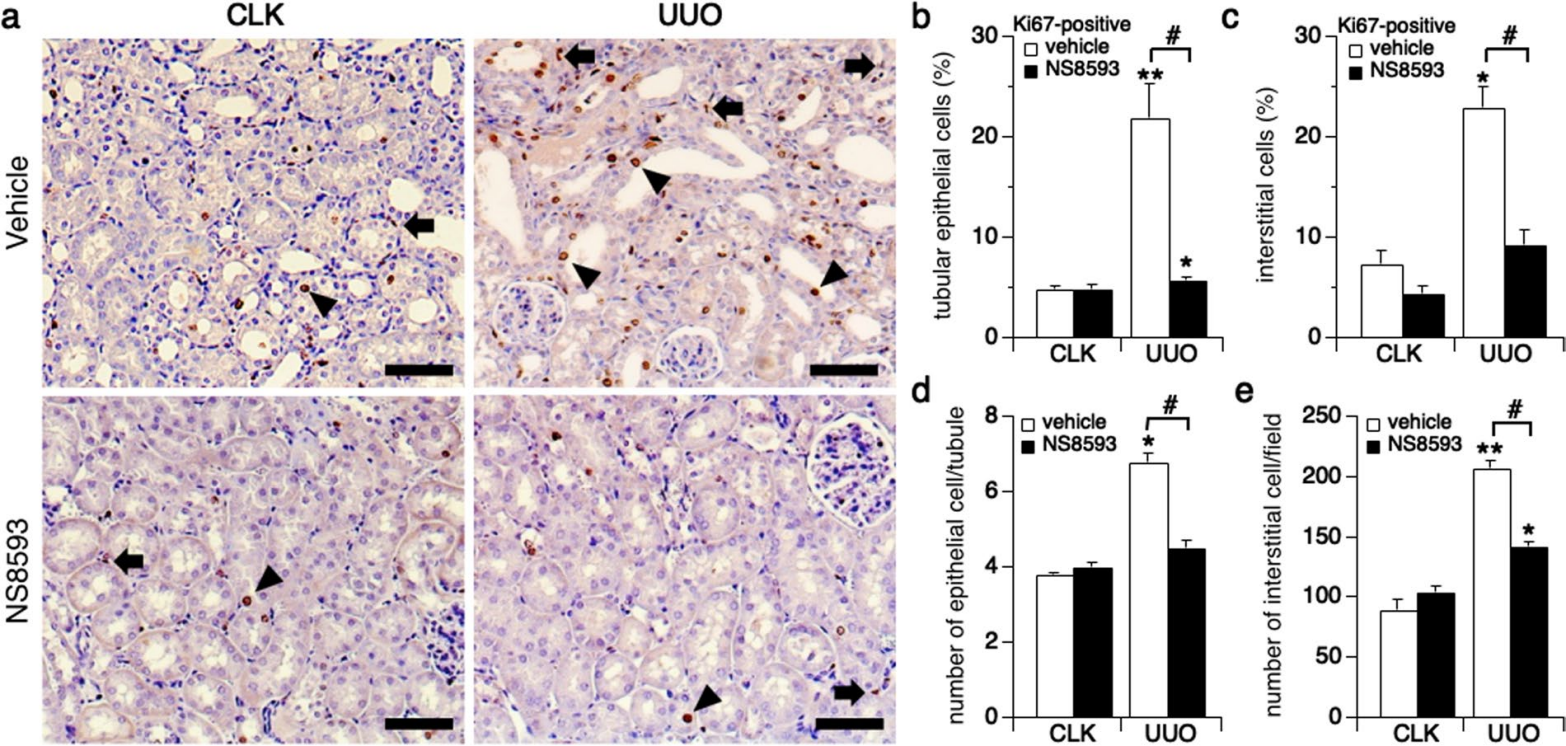
Cell proliferation is suppressed by NS8593 in UUO kidneys. (**a**) Representative pictures of Ki67 immunostaining (magnification x200) from CLK (left panels) and UUO kidneys (right panels) in non-treatment group (upper panels) and NS8593 treatment group (lower panels). Scale bars are 100 μm. Black triangular arrow indicates Ki67-positive tubular epithelial cells. Black arrow points to Ki67-positive renal interstitial cells. (**b**) Quantitative analysis of Ki67-positive renal tubular epithelial cells assessed in 10 representative non-overlapping slides in kidneys from non-treatment (white bars) and NS8593 treatment-groups (black bar), in percent. *p < 0.05 vs. CLK kidney with NS8593 treatment. **p < 0.01 vs. CLK kidney in the non-treatment group, ^#^p < 0.005; UUO kidneys with non-treatment vs. NS8593 treatment. (**c**) The graph plots the percentage of Ki67-positive interstitial cells assessed in 10 slides. *p < 0.005 vs. CLK kidney with non-treatment, ^#^p = 0.001; UUO kidneys with non-treatment vs. NS8593 treatment. (**d**) Average number of epithelial cells counted per tubule in the analyzed field (see also Suppl. Fig. 3 for average number of tubules). *p < 0.001 vs. CLK kidneys with non-treatment, ^#^p < 0.001 UUO kidneys with non-treatment vs. NS8593 treatment. **(e)** The number of total interstitial cells in a field is indicated. *p = 0.005 vs. CLK kidney with NS8593 treatment, **p < 0.001 vs. CLK kidney with non-treatment, ^#^p < 0.001; UUO kidneys in non-treatment vs. NS8593 treatment groups.

TRPM7 is well known to be critically involved in cell proliferation^1^, which is also a hallmark of nephropathy. Our *in vitro* data above show that NS8593 inhibits cell proliferation in both of NRK-52E and NRK-49F cells (Fig. 2). We therefore wondered whether the apparent reduction of highly TRPM7-expressing renal cells seen in UUO kidneys of the NS8593-treatment group could be explained by a change in cell proliferation. To this end we employed the cell proliferation marker Ki67 and immunohistochemistry. Here, the number of both Ki67-positive tubular epithelial and interstitial cells increased significantly in UUO kidneys in the non-treatment group (Fig. 6a–c). In comparison, Ki67 staining was reduced to control levels in UUO kidneys of mice that had received NS8593 treatment (Fig. 6b,c). In analogy, since NS8593 treatment blocked both tubular epithelial and interstitial cell proliferation according to the Ki67 stain, neither the number of epithelial cells per tubule (Fig. 6d) nor the number of interstitial cells (Fig. 6e) increased in UUO kidneys (assessed by analyzing 10 fields). We conclude that NS8593 suppresses tubular cell proliferation induced by kidney damage.

### NS8593 ameliorates renal fibrosis

**Figure 7:**
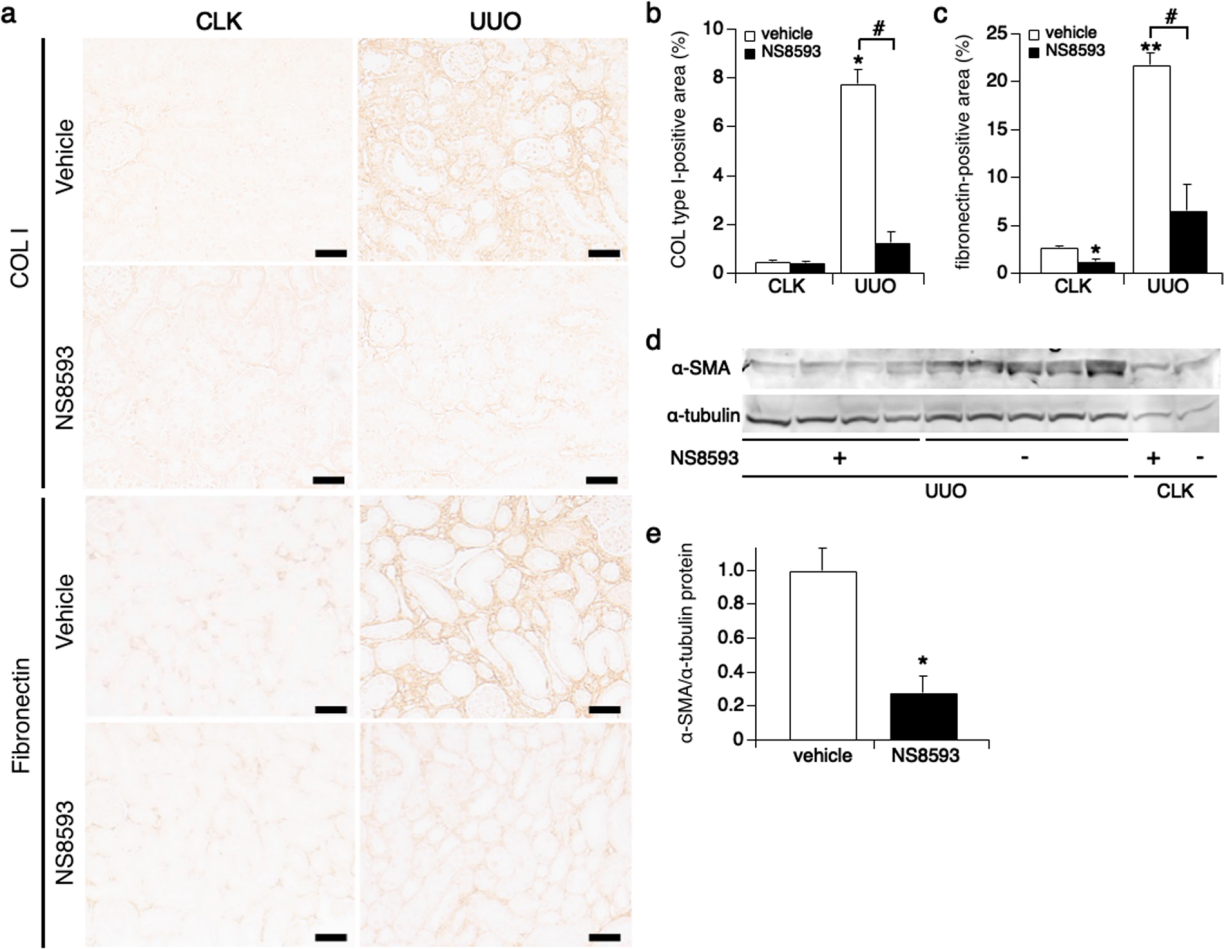
Renal fibrosis is ameliorated by NS8593 treatment. (**a**) Representative pictures of immunostainings for collagen type I and fibronectin in CLK (left panels) and UUO kidneys (right panels, magnification x200) with or without NS8593 treatment. Scale bars are 100 μm. (**b**) The graph plots the average percentage of the collagen type I-positive area in kidneys in non-treatment (white bar) and NS8593 treatment groups (black bar). The staining intensity in the interstitum was computed using ImageJ software. *p < 0.001 vs. CLK kidney with non-treatment, ^#^p < 0.001; UUO kidneys with non-treatment vs. NS8593 treatment. **(c)** The average percentage of fibronectin-positive areas is displayed for CLK and UUO kidneys with or without treatment. *p = 0.001 vs. CLK kidneys with non-treatment, **p < 0.001 vs. CLK kidney with non-treatment, ^#^p = 0.001; UUO kidneys with non-treatment vs. NS8593 treatment. **(d)** The level of α-SMA protein expression was examined in cortical kidney tissue using western blotting. The full-length gel is indicated on Suppl. Fig. S5. **(e)** The intensity of α-SMA protein level computed from western blotting bands in (**d**) using Odyssey CLx Imaging System. The data were normalized by α-tubulin. *p < 0.005 vs. UUO kidneys with non-treatment.

Since NS8593 suppresses both epithelial and interstitial cell proliferation, we asked the question whether this also would lead to a reduced presence of markers of renal fibrosis, namely collagen type I and fibronectin. Using immunohistochemistry with collagen type I and fibronectin antibodies, we quantified the intensity of immunostaining. In non-treated UUO kidneys, collagen type I staining in the tubular interstitium increased to 8% compared to CLK kidneys (Fig. 7a,b; white bars). This effect was completely reversed in the NS8593 treatment group (Fig. 7a,b; black bars). A similar picture emerged for fibronectin (Fig. 7a,c). Furthermore, we confirmed that α-smooth muscle actin (α-SMA), a myofibroblast marker related to renal fibrosis, was significantly decreased in UUO kidneys treated with NS8593 (0.28 vs. 1.0 in UUO kidneys with non-treatment, Fig. 7d,e and Suppl. Fig. S5). Taken together, these results suggest that NS8593 suppresses markers of progressive renal fibrosis following kidney injury.

### The TGF-β1/Smad signaling pathway is suppressed by NS8593 *in vivo*

**Figure 8:**
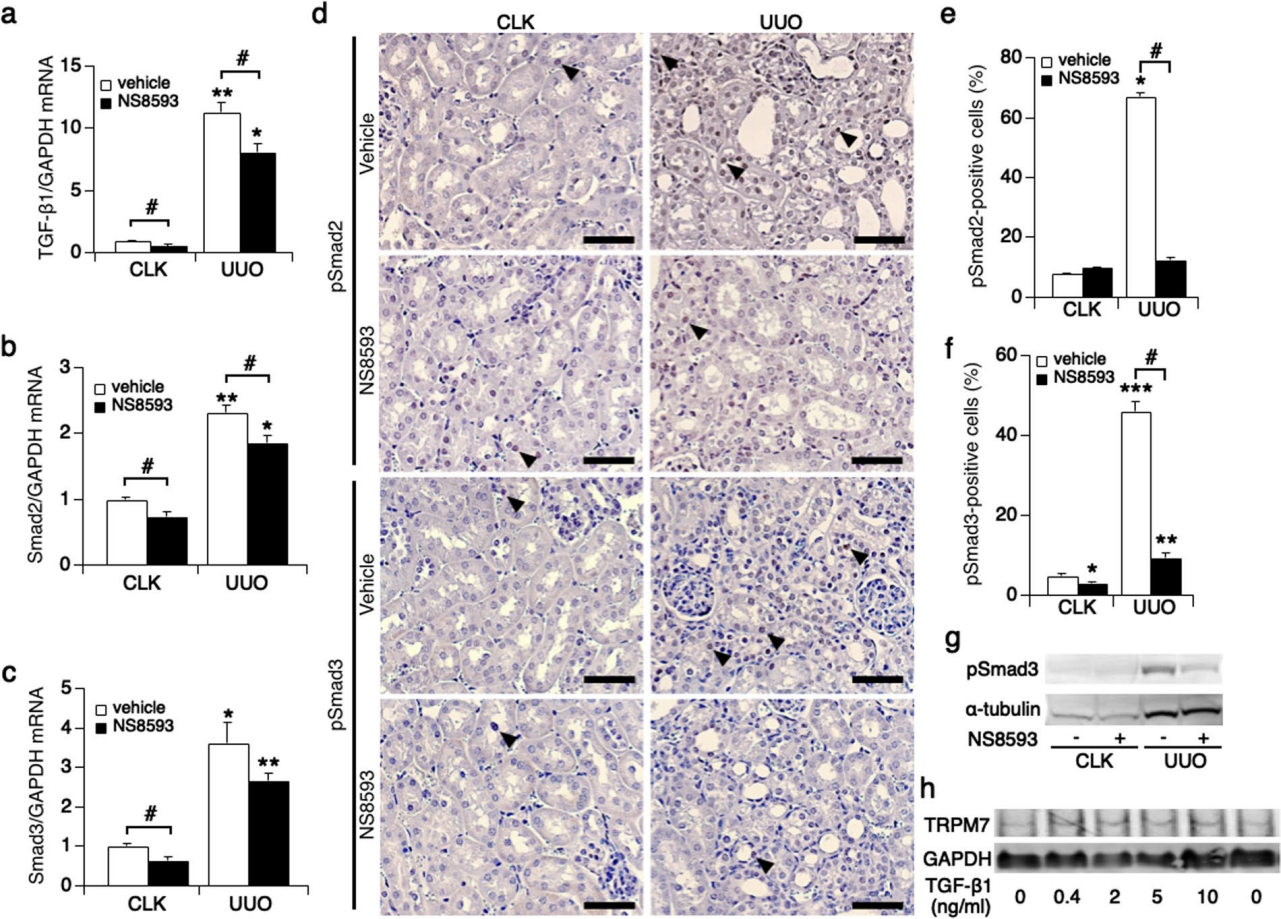
Phosphorylation of Smad2 and Smad3 decreases in UUO kidneys treated with NS8593. mRNA levels of (**a**) TGF-β1, **(b)** Smad2 and **(c)** Smad3 in kidneys of the non-treatment group (white bar) and the NS8593 treatment group (black bar). *p < 0.005 vs. CLK kidneys with NS8593 treatment, **p < 0.001 vs. CLK kidneys with non-treatment in (**a**). *p < 0.01 vs. CLK kidneys with NS8593 treatment, **p < 0.001 vs. CLK kidneys with non-treatment in (**b**). *p < 0.01 vs. CLK kidneys with non-treatment, **p < 0.001 vs. CLK kidneys with NS8593 treatment in (**c**). ^#^p < 0.05 CLK kidneys vs. UUO kidneys. **(d)** Representative immunostaining for Smad2 (pS467) and Smad3 (pS423/pS425) in CLK (left panels) and UUO kidneys (right panels, magnification x200) extracted from mice with or without NS8593 treatment. Scale bars, 100 μm. Triangular arrows point to positively stained tubular epithelial cells. **(e)** Average analysis of phosphorylated Smad2-positive kidney cells in control (white bar) and with NS8593 treatment (black bar), in percent. *p = 0.001 vs. CLK kidneys with non-treatment, ^#^p < 0.001; UUO kidneys with non-treatment vs. NS8593 treatment. **(f)** The average percentage of phosphorylated Smad3-positive cells, *p < 0.05, ***p < 0.001 vs. CLK kidneys with non-treatment. **p < 0.01 vs. CLK kidneys with NS8593 treatment. #p < 0.001; UUO kidneys with non-treatment vs. NS8593 treatment. **(g)** The level of phosphorylated Smad3 protein expression was examined in cortical kidney tissue using western blotting. The full-length gel is indicated on Suppl. Fig. S7. **(h)** The level of TRPM7 protein expression stimulated by TGF-β1 (0, 0.4, 2, 5, 10 ng/ml) examined in NRK-52E kidney cells using western blotting. The full-length gel is indicated on Suppl. Fig. S9.

Activation of TGF-β1/Smad signaling is critically involved in the progression of renal fibrosis^27,29,30^. Previous work has shown that the TRPM7 kinase domain phosphorylates Smad2 and Smad3, thereby activating TGF-β1 signaling in hepatic fibrosis^10^. We therefore assessed whether NS8593 treatment would interfere with Smad2 and Smad3 expression levels in our UUO mouse model (Fig. 8). Indeed, TGF-β1 mRNA level decreased in UUO kidneys of the treatment group in comparison to the non-treatment group (Fig. 8a). Furthermore, Smad2 (Fig. 8b) and Smad3 (Fig. 8c) mRNA expression was suppressed in the treatment group. Since phosphorylated Smad2/3 molecules translocate to the nucleus, we performed immunohistochemistry and evaluated Smad2 and Smad3-positive nuclei in tubular epithelial cells. Phosphorylated Smad2 was significantly reduced in NS8594-treated UUO kidneys (12%) compared to non-treatment (67%, Fig. 8d,e & Suppl. Fig. S6a). Similarly, phosphorylated Smad3 was elevated in UUO kidneys without treatment (46%) and was reduced to 9% with NS8593 (Fig. 8d,f and Suppl. Fig. S6b).

We also performed western blot analyses to assess phosphorylated Smad3 protein levels. The increase in phosphorylated Smad3 observed in untreated UUO kidneys was reduced by NS8593 treatment (Fig. 8g & Suppl. Fig. S7). We next assessed mRNA expression of epithelial mesenchymal transition-(EMT)-related molecules, since phosphorylated Smad2/3 complex translocates to nucleus and then increases transcription of EMT-related molecules to progress renal fibrosis. Similar to protein expression in Fig. 7, the mRNA levels of collagen type I, fibronectin, α-SMA were reduced in UUO kidneys treated with NS8593 compared to non-treatment UUO kidneys (Suppl. Fig. 8a–c). Vimentin is a further fibrotic marker that is regulated by TRPM7 and suppressed by NS8593 following TRPM7 inhibition^31^. We confirmed this reduction of vimentin mRNA in UUO kidneys by NS8593 (Suppl. Fig. S8d). Finally, we stimulated kidney cells with TGF-β1 in order to investigate whether TRPM7 is involved in TGF-β1 signaling in kidney. We confirmed that TRPM7 was up-regulated under TGF-β1 stimulation in NRK-52E cells (Fig. 8h and Suppl. Fig. S9). We conclude that pharmacological manipulation of TRPM7 using NS8593 interferes with Smad2/3 phosphorylation in renal fibrosis.

## Discussion

In this study, we asked the question whether TPPM7 plays a role in renal fibrosis investigating the unilateral ureteral obstruction (UUO) mouse model. We found that TRPM7 is up-regulated in UUO kidneys, particularly tubular epithelial cells. We therefore hypothesized that therapeutic intervention targeting TRPM7 may protect renal tissue from progressive fibrosis. Indeed, using the TRPM7 inhibitor NS8593 suppressed TRPM7 expression and cell proliferation both in tubular epithelial cells and interstitial cells, prevented upregulation of Smad2/Smad3 signaling, and attenuated renal atrophy and fibrosis. These effects, combined with the known inhibitory effects of the compound on both ion channel and kinase function of TRPM7^22,23^ as well as the inhibition of pro-proliferative signaling of macrophages^13,14^, indicate that TRPM7 may represent a promising therapeutic target in kidney fibrosis and TRPM7 inhibitors may act as anti-fibrotic pharmacological tools in this process.

Inhibition of tubular epithelial cell proliferation is an essential component in the prevention of progressive renal fibrosis^17,18,28^. Morphologically, NS8593 treatment reduced the collapse of renal tubules *in vivo* (Fig. 4). This can be explained by the drug inhibiting epithelial cell proliferation and transformation into interstitial cells (Fig. 6). Fewer numbers of interstitial cells will lead to reduced extracellular matrix (ECM) production (Fig. 7), thereby attenuating the progression of kidney fibrosis.

NS8593 indeed suppressed TRPM7 protein expression in unilateral ureteral obstruction (UUO) kidneys, however, not back to the same level as seen in contralateral kidneys (CLK) (Fig. 5). Nevertheless, despite the observation that TRPM7 protein levels remained overall higher than control in the NS8593 treatment group, both cell proliferation and ECM production essentially returned to normal, non-fibrotic levels (Figs. 6 and 7). Since NS8593 interferes with at least one additional ion channel, the SK potassium channel, and perhaps other yet unknown protein targets, additive effects on cell proliferation cannot be excluded when using this compound.

In this study, we verified that TRPM7 antibody from MyBioSource is a specific TRPM7 antibody that detects both human and mouse TRPM7 and can be used for both WB and IHC (Suppl. Fig. S1a,b). While there may be some unspecific bands, the next small band after the predicted band for TRPM7 in Suppl. Fig. S1c is about 50 kDa smaller and likely unrelated to TRPM7. The relevant hTRPM7 control protein isolated from our HEK293-TReX overexpression system tracks well with the predicted TRPM7 band seen in kidney tissue. Since human TRPM7 has 1865 a.a. (212,676 Da) and mouse TRPM7 has 1863 a.a. (212,378 Da), both species have 212 kDa molecular weight, with just 0.3 kDa difference. Hence, we would expect them to indeed locate in a similar location on the WB. These data are highly suggestive that the band indeed represents TRPM7 protein.

During the progression of fibrosis, activated macrophages generate cytokines, including TNFα and TGF-β1, that stimulate renal epithelial cell proliferation as well as production of ECM by myofibroblasts. Previous work has shown that NS8593 abolishes proliferation and polarization of macrophages via TRPM7 inhibition^13^. In addition, TRPM7 is critical in mediating the cytosolic Ca^2+^ elevations essential for LPS-induced macrophage activation and nuclear translocation of NFκB^14^. Thus, NS8593 might lead to a decrease in cytokine release due to the inhibition of macrophage activation and proliferation in our UUO mouse model. A decrease in cytokine production would then reduce kidney cell proliferation and with this reduce ECM production in nephropathy.

Variations of intracellular calcium (Ca^2+^) concentrations play a critical role in cellular signaling, and regulate signal transduction, transcription, cell proliferation and apoptosis. Store-operated calcium entry (SOCE) represents one major mechanism in this process. SOCE is critically involved in cytokine production through calcium-release activated Ca^2+^ (CRAC) channels via STIM1, STIM2 and Orai1^3,23,32,33^. Furthermore, SOCE activity is facilitated by TRPM7 via its kinase function^23,34^. The strongly enhanced expression level of TRPM7 in UUO kidneys might therefore not only lead to enhanced tubular epithelial cell proliferation, but also to enhanced SOCE, and with this, enhanced cytokine-production and aggravation of fibrosis. NS8593 is known to act both on TRPM7 channel function as well as TRPM7 kinase activity^22,23^. While NS8593 does not interfere with SOCE directly, the drug suppresses SOCE through inhibition of the TRPM7 kinase domain^23^. This would lead to a suppression of cytokine production. The drug’s interference with the regulatory effect of TRPM7 on SOCE might therefore be a contributing factor in attenuating the progression of renal fibrosis.

Administration of NS8593 *in vivo* could lead to perturbances in the systemic divalent cation homeostasis due to suppression of TRPM7-dependent uptake of minerals in the colon, as has previously shown for waixenicin A, a natural product inhibitor of TRPM7 derived from Sarcothelia edmonsoni^35^. It will be interesting to see whether the protective effect of TRPM7 suppression in kidney fibrosis is mainly due to TRPM7 channel or kinase activity itself, a likely hypomagnesemia induction (or divalent perturbance), or a combination thereof.

Studies using UUO and other kidney injury mouse models have established that the TGF-β/Smad transduction is a central contributing factor in renal fibrosis^29,30^. Here, phosphorylated Smad2/3 is translocated to the nucleus and upregulates transcription of target genes that lead to cell proliferation and production of ECM. Importantly, treatment with anti-TGF-β antibody ameliorates progressive kidney damage via inhibition of TGF-β/Smad signaling pathway^27^. In hepatic and cardiac fibrosis, TRPM7 is upregulated by TGF-β1 stimulation and Smad2/3 phosphorylation^10,36^. In addition, a recent report showed that Smad2 is a substrate of the TRPM7 kinase^3,37^. Thus, TRPM7 upregulation serves as a positive feedback loop to Smad signaling, thereby further aggravating fibrosis^10^. Our data are aligned with these findings in that NS8593 suppresses Smad2/3 signaling (Fig. 8), which in turn then may contribute to the reduced renal cell proliferation (Fig. 6) and ECM production (Fig. 7) thereby protecting kidneys from renal injury and fibrosis.

In summary, TRPM7 may represent a nexus for a variety of cellular events that all contribute synergistically to pro-inflammatory and pro-proliferative mechanisms that ultimately result in kidney fibrosis. These include TRPM7’s own divalent cation-conducting channel activity, its kinase activity that can contribute to both proliferation as well as other Ca^2+^ mobilization mechanisms such as SOCE, and Ca^2+^-dependent production of pro-inflammatory and pro-proliferative cytokine production. Changes in TRPM7 expression or activity can shape the fibrotic progression of kidney disease and pharmacologic interference with TRPM7 expression and/or function may offer therapeutic benefits to treat this debilitating condition.

## Materials and Methods

### Experimental animals and design of the UUO mouse model

Male C57Bl/6 mice 6 weeks of age, weighing 20 to 25 g at the start of the experiment, were purchased from Charles River Laboratories (USA) and allowed free access to food and water. Unilateral ureteral obstruction (UUO) was created by ligating the left ureter with 3–0 silk through a left lateral incision under anesthesia of isoflurane^17,28,30^. NS8593 was purchased from Sigma (USA) and kept as a stock solution of 100 mM dissolved in DMSO. NS8593 was diluted in saline (1 mg/ml) before injection. UUO mice in the treatment group were administered 5 mg/kg of NS8593 daily via intraperitoneal (i.p.) injection (n = 4), with the first injection starting directly after surgery. This dose has previously been found adequate in animal models to reduce atrial fibrillations in rats and dogs^38,39^. The non-treatment group received DMSO, appropriately diluted in saline, i.p. injected daily (n = 5). Mice were sacrificed using inhalation of 2% isoflurane on day 7 after surgery. Obstructed kidneys (UUO), non-obstructed contralateral kidneys (CLK), blood was collected and subjected to the experiment described below. The experimental procedures were reviewed and approved by the Institutional Animal Care and Use Committee of the University of Hawaii and the Animal Care Committee at The Queen’s Medical Center. All procedures were in accordance with guidelines recommended by the NIH.

### RNA isolation and qRT-PCR

Total RNA was extracted from cortical kidney tissues using RNeasy Protect Mini Kit (Qiagen, USA) according to manufacturer’s instructions. Reverse transcription of the RNA was performed using the SuperScript ΙΙΙ First-Strand Synthesis System for RT-PCR kit (Invitrogen, USA). The resulting cDNA was subjected to real-time PCR using the iCycler iQ 5 (Bio-Rad Laboratories, USA) for amplification and online quantification. All PCR experiments were performed using PrefeCTa SYBR Green SuperMix for iQ (Quanta biosciences, USA). The primer sequences for mouse TRPM7 were sense 5′-GGAACAGGCTATGCTTGATGC-3′ and anti-sense 5′-CATTGGATTGGTTGGACCTTG-3′, giving an amplified RT-PCR product of 148 bp. The primer sequences used were: TGF-β1 sense 5′-CCTGAGTGGCTGTCTTTTGGACG-3′ and anti-sense 5′-ATGGAGCGCTGAATCGAAAGC-3′ (91 bp), Smad2 sense 5′-CCAGGTCTCTTGATGGTCGT-3′ and anti-sense 5′-GGCGGCAGTTCTGTTAGAAT-3′ (252 bp), Smad3 sense 5′-ACACATTGGGAGAGGTGTGC-3′ and anti-sense 5′-GCAAGGGTCCATTCAGGTGT-3′ (349 bp). Primers for GAPDH used as an internal control were sense 5′-TGCACCACCAACTGCTTAG-3′ and anti-sense 5′-GATGCAGGGATGATGTTC-3′, giving an amplified RT-PCR product of 176 bp. Real-time RT-PCR data were analyzed according to the manufacturer’s instructions, the ratio of TRPM7 mRNA was normalized by GADPH mRNA in each sample.

### Western blot assay

Cortical kidney tissues were homogenized in radioimmunoprecipitation assay (RIPA) buffer (25 mM Tris-HCI pH7.4, 150 mM NaCl, 0.1% SDS, 0.5% Triton-X100, 0.5% sodium deoxycholate) containing protease inhibitor cocktail (Sigma-Aldrich, USA) for 30 mins at 4 °C. After incubation, lysates were centrifuged at 13,000 rpm for 15 mins at 4 °C and protein concentrations of lysates were measured using a protein assay reagent (Bio-Rad Laboratories)^16,27^. Soluble lysates were boiled with NuPAGE LDS sample buffer (Invitrogen) at 98 °C for 5 min. Equal amounts of proteins (80 μg for TRPM7 and 35 μg for α-SMA and pSmad3) were loaded and separated in NuPAGE 3–8% gel (Invitrogen) then transferred to PVDF membrane (Millipore, USA). Proteins were detected using the antibodies of rabbit polyclonal anti-human TRPM7 (MyBioSource, USA, MBS89214600), mouse monoclonal anti-human α-SMA (Dako, CA, USA), anti-GAPDH (6C5, Abcam, UK), anti-human α-tubulin (DM1A, Novus Biologicals, CO, USA), rabbit monoclonal anti-human pSmad3 (phosphor S423 + S425, Abcam) followed by treatment with IRDye infrared fluorescence-conjugated anti-rat and anti-mouse secondary antibodies (LI-COR, USA). The antibody-bound proteins were visualized and quantified using Odyssey CLx Imaging System (LI-COR). GAPDH and α-tubulin were used as an internal control. TRPM7 and α-SMA intensities were normalized to internal control in each sample.

### Cell culture

The normal rat kidney fibroblast cell line NRK-49F and epithelial cell line NRK-52E were purchased from ATCC (USA). These cells were maintained in DMEM medium (ATCC) supplemented with 5% fetal calf serum (ATCC) at 37 °C in 95% air and 5% CO_2_ atmosphere.

### Fluorescent 96-well kinetic plate reader assay

A 96-well fluorescent kinetic plate reader (Hamamatsu FDSS-7000EX, Hamamatsu Photonics KK. Japan) was used to investigate the inhibitory efficacy of NS8593 on kidney cells. NRK-49F and NRK-52E cells (50,000 cells/well) were plated in poly-L-lysine-coated black 96 well plates with clear bottom (Greiner, Austria). The culture medium was removed the next day, replaced with fresh culture medium supplemented with 2 μM Fura-2AM (Invitrogen), 0.1% Pluronic F-127 (Sigma-Aldrich) and 2 mM Probenecid (Sigma) and incubated at 37 °C under 5% CO_2_ incubator for 1 hour. The plates were washed 3 times with solution containing (in mM): 140 NaCl, 2.8 KCL, 2 MgCl_2_, 1 CaCl_2_, 10 HEPES-NAOH, 11 Glucose (pH 7.2, 300 mOsm) after Fura-2AM incubation, fresh solution added, and transferred to FDSS-7000EX. Baseline fluorescence was measured for 1 min before application of NS8593 (0, 0.1, 0.3, 1, 3, 10, 30 μM respectively). After 3 min incubation with NS8593, 0.5 mM MnCl_2_ was applied. Data acquisition continued for another 4 min. Fluorescent signals were assessed at an excitation wavelength of 360 nm, and emission was measured at 510 nm to visualize intracellular Mn^2+^ quench of Fura-2. Data were normalized to the point before application of MnCl_2_, and Mn^2+^ quench was measured at 480 s into the experiment. The 0 μM NS8593 vehicle-control trace was set as maximum quench (100%).

### Cell proliferation assay

Cell viability was examined using the MTT assay. NRK-52E and NRK-49F were seeded at a density of 3,000 cells into 96-well plates with media. NS8593 (0, 0.1, 0.3, 1, 3, 10, 30 μM) was added to individual wells. Plates were incubated at 37 °C and 5% CO_2_. NRK-52E cells were assessed 2 days after plating and incubation. For NRK-49F, the cell culture medium was replaced after 24 hours and then once daily with fresh NS8593 for a total of 3 days. After 3 days, the culture medium was removed and 65 μl of 500 μg/ml thiazolul blue tetrazolium bromide (MTT, Sigma) in DMEM was added to each well. Cells were incubated at 37 °C under 5% CO_2_ for 1 hour and checked for purple crystal formation. Cells were washed twice with PBS (Gibco, USA) and 200 μl DMSO was added as MTT solvent. Absorbance was assessed at 535 nm using a Benchmark Plus microplate spectrophotometer (Bio-Rad Laboratories). Data were normalized to control (0 μM NS8593). All experiments were performed in triplicate.

### Electrophysiology

Patch clamp experiments were performed in the whole-cell configuration^40^. Currents were elicited by a ramp protocol from −100 mV to +100 mV over 50 ms acquired at 0.5 kHz and a holding potential of 0 mV. Inward current amplitudes over the course of the experiment were extracted at −80 mV, outward currents at +80 mV and plotted versus time. Data were normalized to cell size measured immediately after whole-cell break-in (pA/pF). Capacitance was measured using the automated capacitance cancellation function of the EPC-9 (HEKA, Germany). All values were given as mean ± standard error of mean (S.E.M). Patch pipettes (Sutter Instrument, CA, USA) were pulled and polished, had a tip resistance of 2–3 MΩ when filled with internal solution. Standard extracellular solution contained (in mM): 140 NaCl, 2.8 KCl, 2 MgCl_2_, 1 CaCl_2_, 10 HEPES-NaOH, 11 Glucose (pH 7.2, 300 mOsm). Intracellular solution contained (in mM): 140 K-glutamate, 8 NaCl, 10 K-BAPTA, 10 HEPES-KOH (pH 7.2, 300 mOsm). NS8593 (Sigma) was dissolved in DMSO as 10 mM stock solution and stored at −20 °C, diluted to 10 μM with the extracellular solution used for patch-clamp experiments. As a control application, DMSO was diluted to same concentration of diluted NS8593 in extracellular solution.

### Immunohistochemical analysis

Kidney tissues were fixed in 4% paraformaldehyde in phosphate-buffered saline overnight and embedded in paraffin. The immunoreactivities for TRPM7, Ki67, collagen type Ι, fibronectin, pSmad2 and pSmad3 were determined using a VECTASTAIN ABC kit (Vector laboratories, USA) as a standard biotin-streptavidin-peroxidase method. 3 μm-thick kidney tissue sections were deparaffinized and rehydrated, then antigen retrieval was performed in 10 mM citrate buffer (pH 6.0) using a microwave for 15 mins. Endogenous peroxidase activity was blocked in 3% hydrogen peroxide for 10 mins. The primary antibodies were as follows: rabbit polyclonal anti-human TRPM7 (MyBioSource, MBS89214600, dilution 1:100), anti-human Ki67 (Abcam, 1:1000), anti-human collagen type Ι (Abcam, 1:350), anti-human fibronectin (Abcam, 1:500), anti-human Smad2 (phospho S467, Abcam, 1:50) and rabbit monoclonal anti-human Smad3 (phosphor S423 + S425, Abcam, 1:200). The secondary antibodies were affinity-purified biotinylated goat anti-rabbit immunoglobulin with ImmPACT DAB kit (Vector laboratories) as peroxidase substrates. The kidney sections were counterstained with hematoxylin. TRPM7-positive epithelial cells were binned into high expression level (strongly TRPM7-positive cells), low expression level (slightly TRPM7-positive cells) and no detectable TRPM7 expression. The number of TRPM7 and Ki67-positive tubular epithelial and interstitial cells were counted in 10 randomly selected, non-overlapping renal cortical fields at x200 magnification, and the mean values were obtained. The number of tubules and tubular epithelial cells were counted using the kidney sections stained for Ki67. The number of renal tubular epithelial cells in a tubule was determined as the total number of tubular epithelial cells in one field, divided by the number of total tubules. The collagen type Ι and fibronectin-positive interstitial areas were quantified in 10 randomly selected, non-overlapping renal cortical fields at x200 magnification using ImageJ software. We set a threshold to compute the positive areas for each field and computed the ratio of the positive areas over the whole interstitial area.

### Histopathological staining

3 μm-thick kidney tissue sections were rehydrated and stained with Masson’s trichrome for histopathological analysis. As previously described^17,28^, the levels of fibrotic tubulointerstitial lesions, tubule dilation, tubular atrophy and interstitial fibrosis were graded semi-quantitatively as follows: 0, absent (0%); 1, weak (≤20%); 2, mild (21–50%); 3, moderate (51–80%); 4, severe (≥80%) in whole cortical fields of kidney at X200 magnification. The average of grading of each mouse was calculated and used for statistical analysis.

### Statistical analysis

All values were given as means ± SEM. Differences between groups were examined for statistical significance by analysis of variance (ANOVA). Values of p < 0.05 were considered as statistically significant.

### Ethical standards

The experiments comply with the current laws of the United States of America, where they were performed.

## Supplementary information

Supplementary Information.
