# Supplementary material for: TRPM7 contributes to progressive nephropathy
Source: Sci Rep. 2020 Feb 11;10:2333. doi: 10.1038/s41598-020-59355-y (PMC7012919; doi:10.1038/s41598-020-59355-y)
Supplement: Supplementary file 1 — Supplementary Information. [file 41598_2020_59355_MOESM1_ESM.pdf]

TRPM7 contributes to progressive nephropathy.

Sayuri Suzuki[1,3], Reinhold Penner[1,2,3], Andrea Fleig[1,2,3]

[1]Center for Biomedical Research, The Queen's Medical Center, 1301 Punchbowl St., Honolulu, HI 96813, U.S.A.

[2]University of Hawaii Cancer Center, University of Hawaii, 651 Ilalo St., Honolulu, HI 96813

[3]John A. Burns School of Medicine, University of Hawaii, 651 Ilalo St., Honolulu, HI 96813

SUPPLEMENTARY METHODS

Cell culture

Tetracycline (Tet)-inducible HEK293-TREx cells stably transfected with HA-tagged human TRPM7 wild type (hTRPM7), were cultured in DMEM medium (Sigma, USA) containing 10% fetal bovine serum (FBS) (Corning, USA), blasticidin (5 µg/ml) (Gibco, USA), and zeocin (0.4 mg/ml) (Gibco). Overexpression was induced adding 1 µg/ml tetracycline (Gibco) to the culture medium. Cells were collected before- and 15 hours after tetracycline induction.

Western blot assay and blocking activity of the TRPM7 antibody

Harvested TRPM7 overexpressing HEK293-TReX cells were dissolved in RIPA buffer and 50 µg protein was loaded and separated in NuPAGE 8% gel and then transferred to the PVDF membrane as the kidney tissue samples. Proteins were detected using the antibodies of rabbit polyclonal anti-human TRPM7 (MyBioSource, #MBS89214600). A specific TRPM7 blocking peptide (MyBioSource, MBS9227494) was used to block the activity of the TRPM7 antibody (MyBioSource, #MBS89214600). TRPM7 blocking peptide and TRPM7 antibody were mixed and incubated for 1 hour at room temperature then used for immunoblotting. After detection of protein using the Odyssey CLx Imaging System, TRPM7 antibody-blocking peptide complexes were stripped from the membrane in stripping buffer (1M Tris-HCl (pH6.8), 10% SDS, 1% β-melcapto ethanol) and re-probed with TRPM7 antibody.

Immunohistochemical analysis and blocking activity of the TRPM7 antibody in mouse kidney tissue

The immunoreactivities for TRPM7 were determined with TRPM7 antibody (MyBioSource, #MBS89214600) using VECTASTAIN ABC kit. To confirm the specificity of TRPM7 antibody, TRPM7 blocking peptide (MyBioSource, MBS9227494) and TRPM7 antibody were mixed and incubated for 1 hour at room temperature then applied to kidney tissue sections. After TRPM7 antibody incubation, the immunohistochemistry processing was followed as instructed by the manufacturer (VECTASTAIN ABC kit).

RNA interference

The small interfering RNA (siRNA) specific against rat TRPM7 (XM_001056331) and control siRNA (SIC001_10NM0L) were purchased from Sigma. siRNA transfection was performed using Lipofectamine2000 (Invitrogen) according to the manufacturer's protocols on NRK-52E and NRK-49F cells. After 24 h of siRNA treatment, cells were re-plated 3 x 10^3 cells on 96-well plates for MTT assay.

qRT-PCR

Real-time RT-PCR was performed using the following primer sequences:
Collagen type I: sense 5'-AGAGCATGACCGATGGATTCC-3' and anti-sense 5'-TTGCCAGTCTGCTGGTCCATG-3'
Fibronectin: sense 5'-ACGGTTTCCCATTACGCCAT-3' and anti-sense 5'- CTTTCCATTCCCGAGGCAT-3'
α-SMA: sense 5'-ACTGGGACGACATGGAAAAG-3' and anti-sense 5'-CATCTCCAGAGTCCAGCACA-3'
Vimentin: sense 5'-AGAGGAAGCCGAAAACAC-3' and anti-sense 5'-AAGGGCATCCACTTCACAG-3'

Suzuki et al.

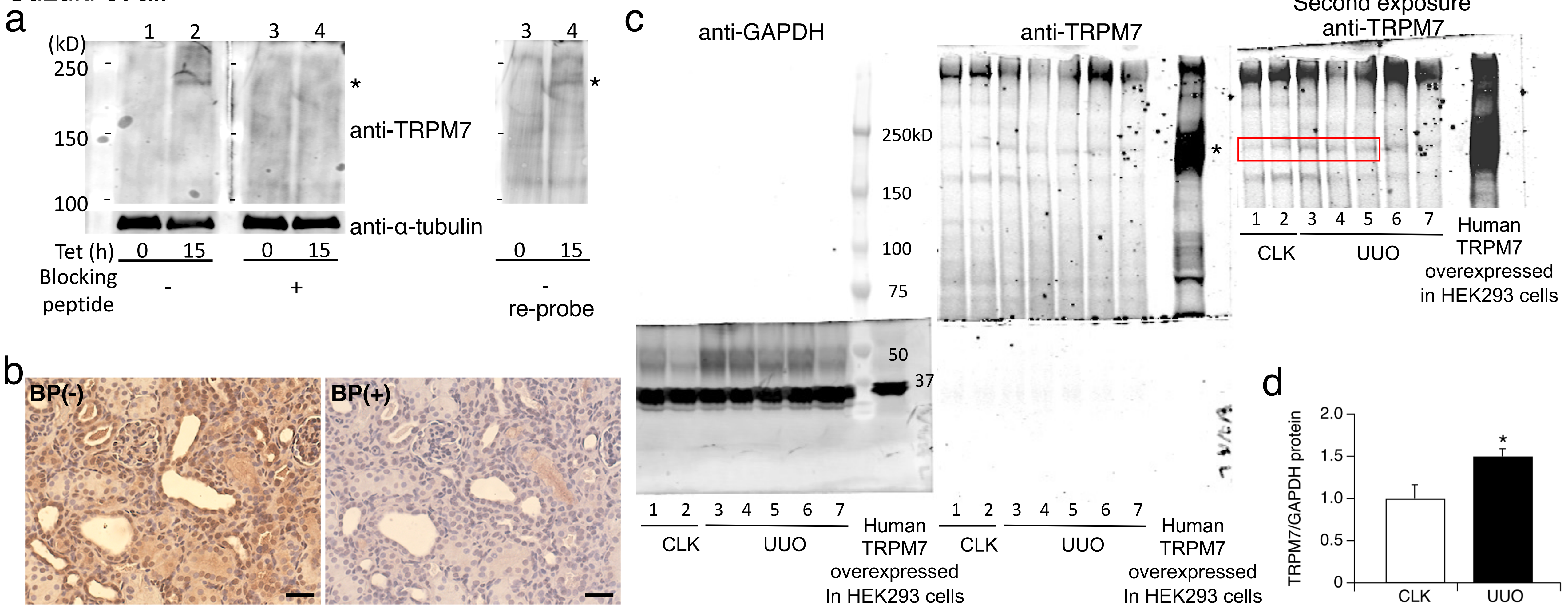

Supplementary Figure S1: The expression of TRPM7 protein is up-regulated in renal damage.

(**a**) The specificity of the commercial TRPM7 antibody (see methods) was confirmed by western blot using cell lysates derived from HEK293 cell with (15 hrs) or without (0 hrs) tetracycline induction of human TRPM7 protein. Immunoblotting was performed with TRPM7 antibody (lane1,2). TRPM7 blocking peptide was used for blocking the activity of TRPM7 antibody (lane 3,4). The membrane (lane 3,4) was re-probed with TRPM7 antibody in absence of blocking peptide. Asterisk marks the hTRPM7 band. (**b**) The specificity of the TRPM7 antibody on immunostaining in mouse kidney tissue. Immunohistochemistry of TRPM7 was performed without (left panel) or with TRPM7 blocking peptide (right panel) using UUO kidneys. Immunostaining was strongly reduced when using the TRPM7 blocking peptide. **(c)** The full-length gel of WB in Fig.1b. The data in Fig.1b was cropped from second exposure gel (right gel, red square). Asterisk marks the TRPM7 band. **(d)** The intensity of TRPM7 protein level computed from WB bands (Fig. 1b) using the Odyssey CLx Imaging System. The data were normalized by GAPDH. *$p < 0.05$ vs. CLK kidneys.

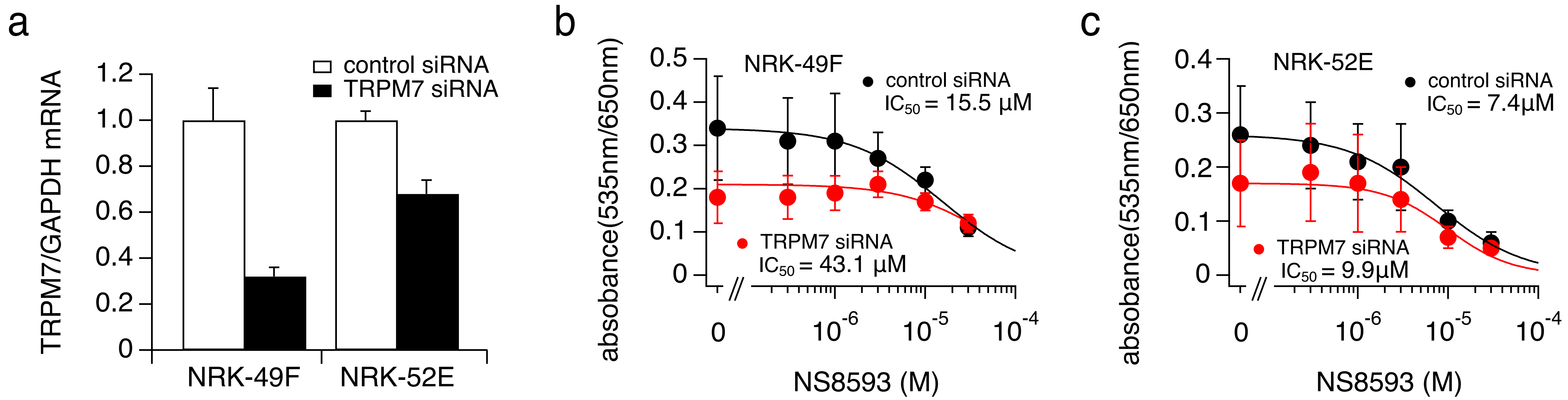

Supplementary Figure S2: siRNA-mediated knockdown of TRPM7 shifts the IC_{50} of NS8593-induced inhibition of cell growth.

(a) The level of TRPM7 mRNA in NRK cells after siRNA treatment was indicated as the ratio of TRPM7/GAPDH. White bars represent control siRNA treatment and black bars represent TRPM7 siRNA treatment. **(b)** The inhibitory effect of NS8593 on cell proliferation was examined in NRK-52E cells using MTT assays. After 24 h of siRNA treatment, cells were re-plated on 96-well plates and started to incubate with each concentration of NS8593 for 2 days. Data were averaged, plotted and approximated with a dose-response fit (n = 3). **(c)** Same MTT experiment as in (b) but with NRK-49F cells (n = 3). Here, cells were incubated with increasing concentrations of NS8593 for 3 days before the MTT analysis. The plate medium was exchanged every 24 hours with fresh medium supplemented with appropriate NS8593 concentrations.

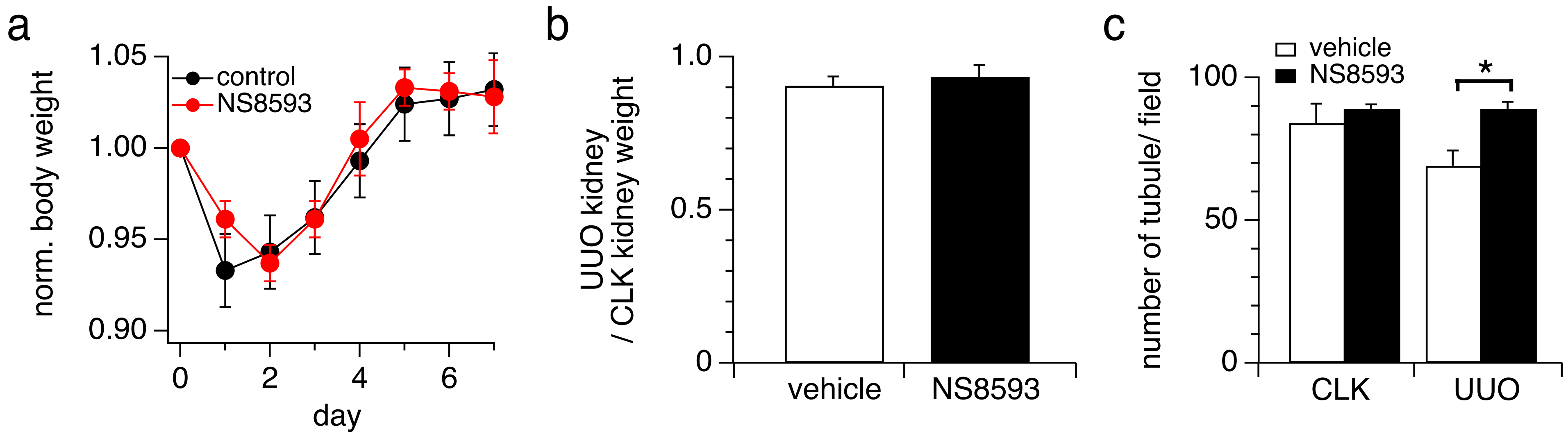

Supplementary Figure S3: NS8593 prevents kidney atrophy from renal damage.

(a) Body weight of mice was measured daily before injection. Body weight of each day was normalized by the weight before surgery and the average is indicated (n = 4-5). Black circles represent the non-treatment control group and red circles are the NS8593 treatment group. **(b)** The weight of UUO kidney at day 7. Each UUO kidney weight was normalized to the weight of the corresponding CLK kidney. **(c)** The total number of renal tubules assessed in one representative field in non-treatment (white bar) and NS8593 treatment groups (black bar). *$p < 0.05$; UUO kidneys in non-treatment vs. NS8593 treatment. **(d)** Quantification of serum creatinine levels.

Suzuki et al.

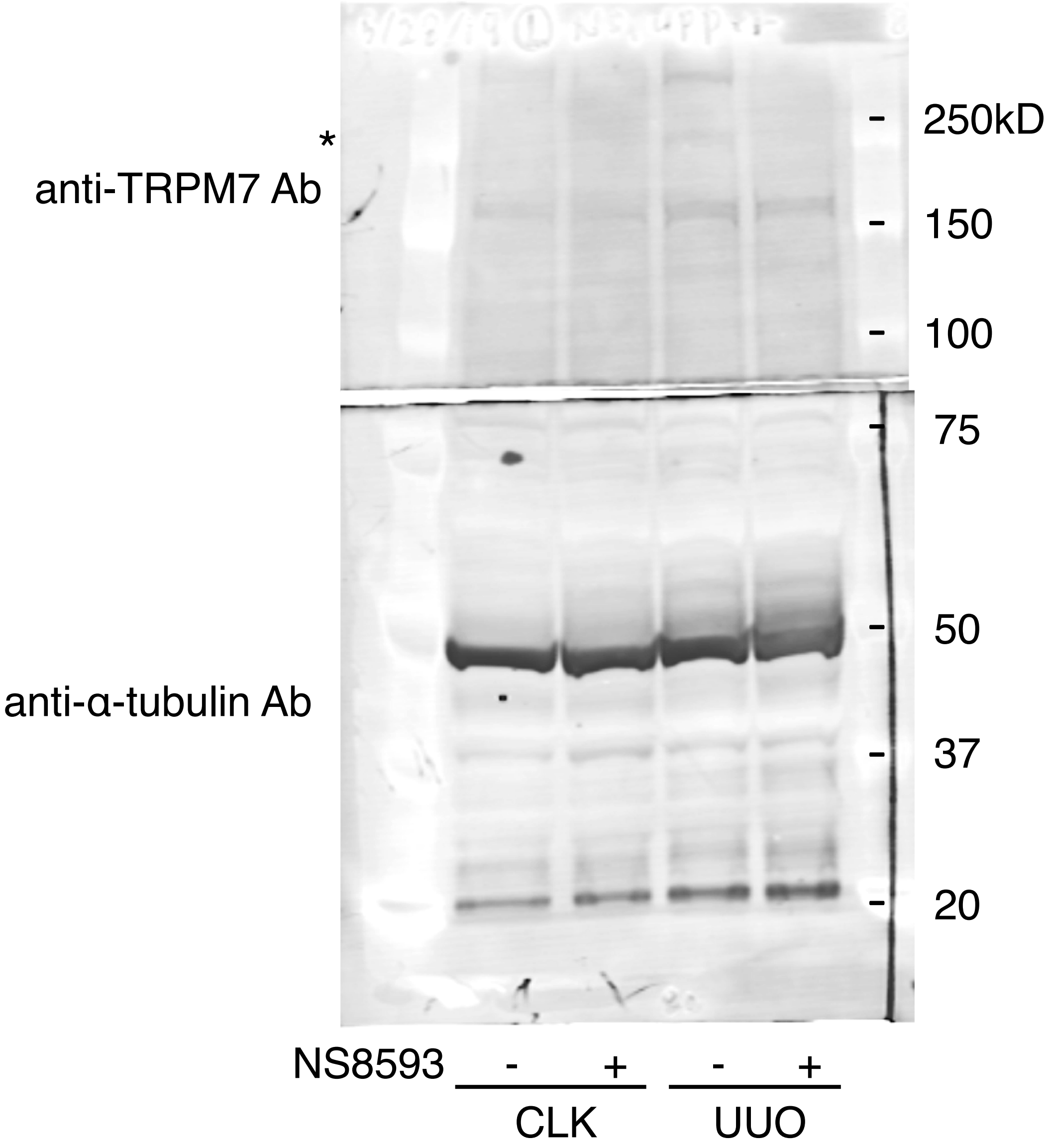

Supplementary Figure S4: The expression of TRPM7 protein in renal damage is reduced by NS8593.

Western blot was performed using cortical kidney tissues from UUO mice. Increased TRPM7 protein in UUO kidneys was reduced by NS8593 treatment. Asterisk marks the TRPM7 band.

Suzuki et al.

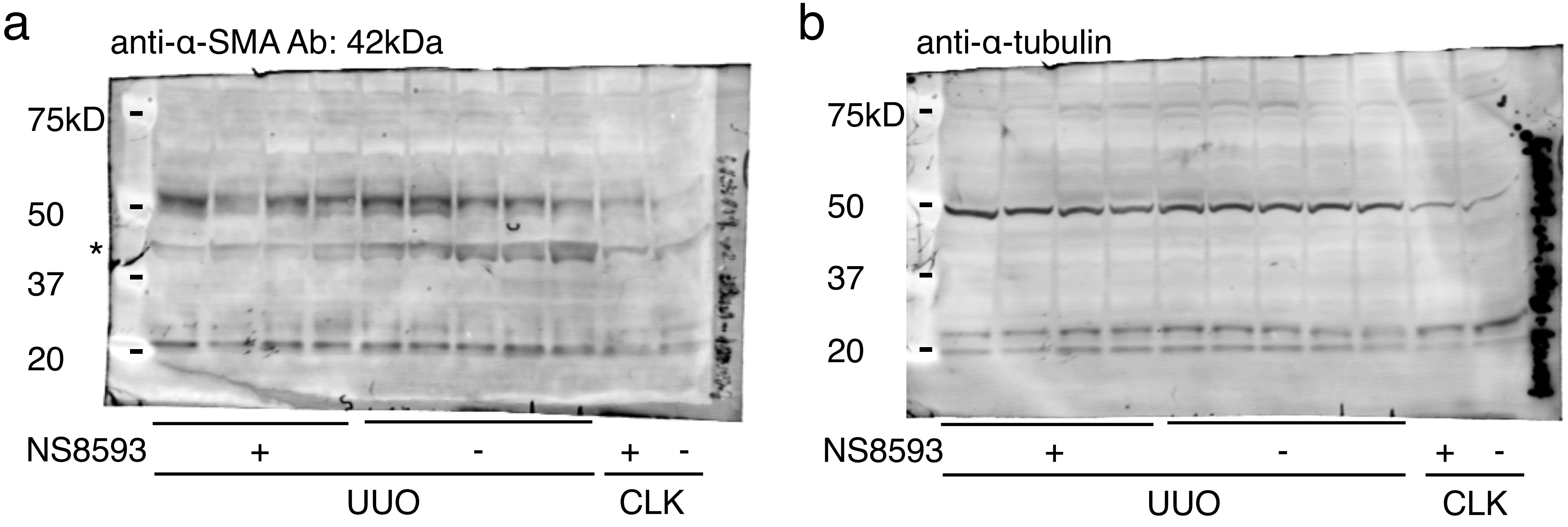

Supplementary Figure S5: α-SMA protein in UUO kidneys is decreased by NS8593.

The full-length western blot of **(a)** α-SMA and **(b)** α-tubulin shown in Fig. 7d. Asterisk marks the α-SMA band in (a).

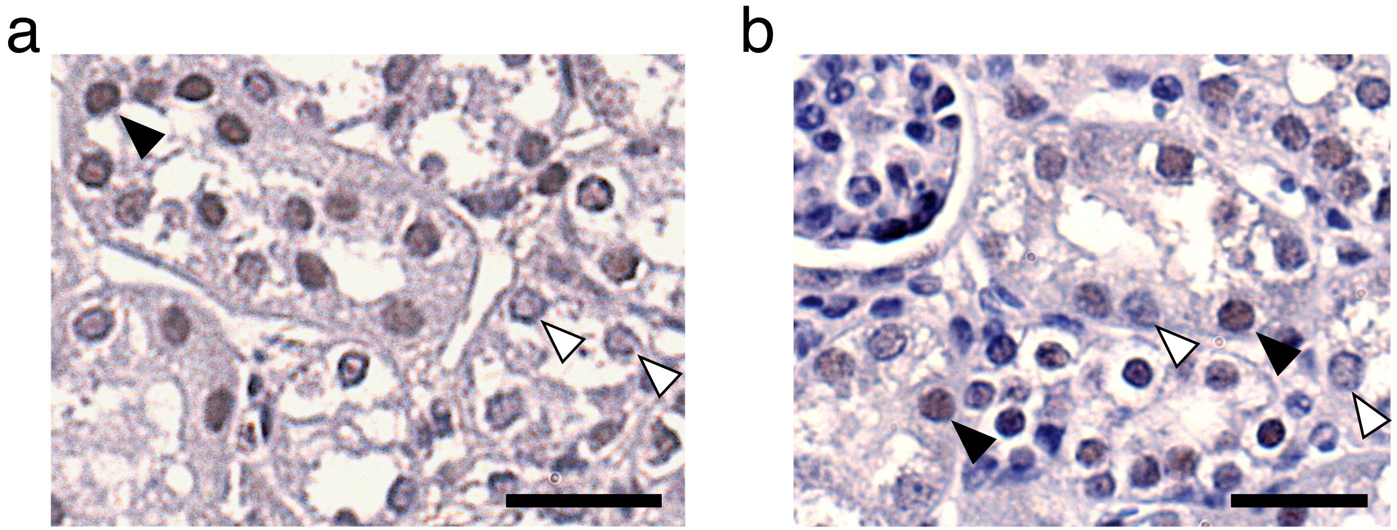

Supplementary Figure S6: Immunohistochemistry using anti-pSmad2 and anti-pSmad3 antibodies.

High magnification pictures of (**a**) pSmad2 and (**b**) pSmad3 immunostaining in UUO kidneys with non-treatment were extracted from Fig. 8d. Black triangular arrows point to positively stained and white triangular arrows indicate negatively stained tubular epithelial cells. Scale bars = 50 μM.

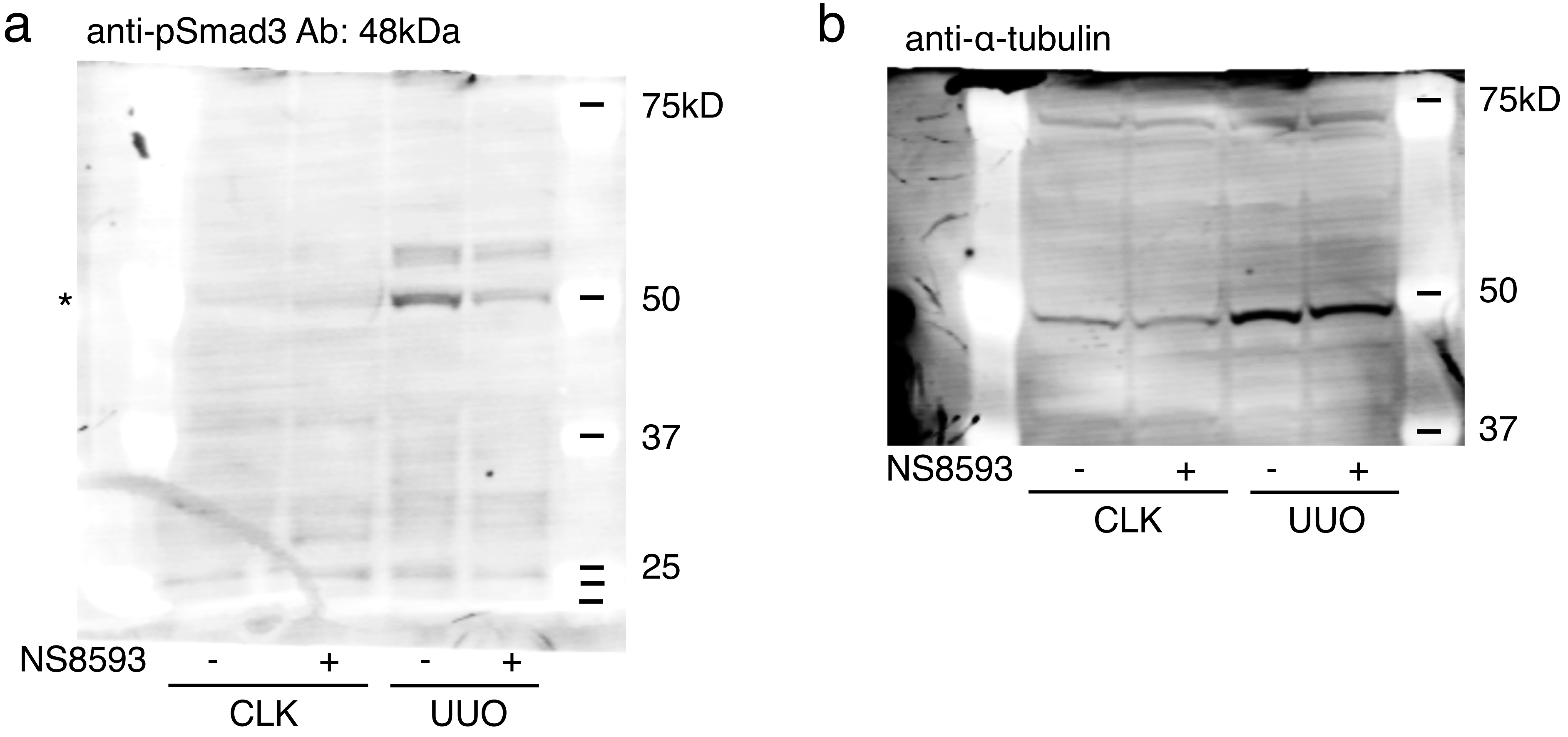

Supplementary Figure S7: pSmad3 protein in UUO kidneys is decreased by NS8593.

The full-length western blots of **(a)** pSmad3 and **(b)** α-tubulin in Fig. 8g. Asterisk marks the pSmad3 band in (a).

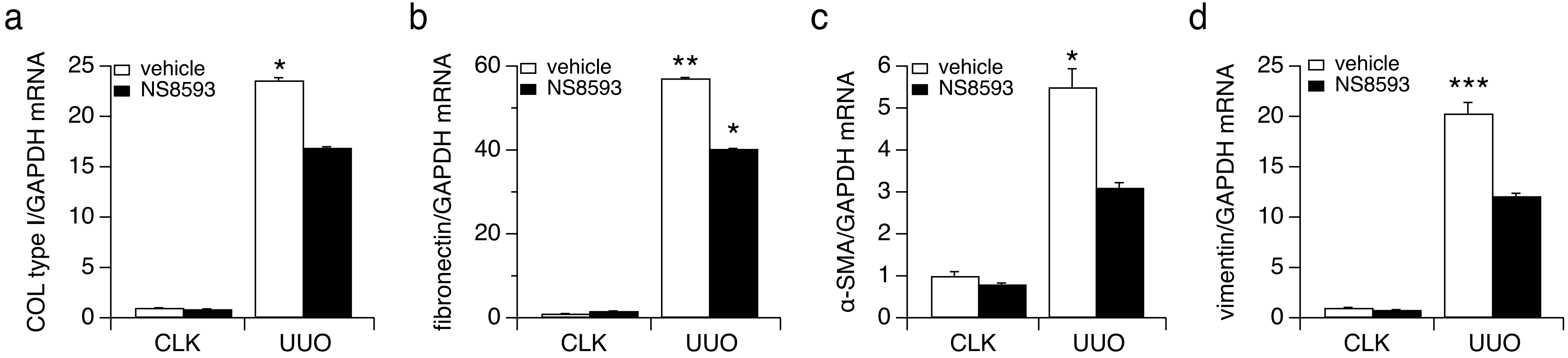

Supplementary Figure S8: NS8593 treatment suppressed transcription of EMT-related molecules induced by TGF-β1/Smad signaling pathway.

The panels illustrate mRNA levels of **(a)** collagen type I, **(b)** fibronectin, **(c)** α-SMA, **(d)** vimentin. White bars represent non-treatment groups and black bars represent NS8593 treatment groups. $^{*}p < 0.05$, $^{**}p = 0.01$, $^{***}p < 0.001$ vs. CLK kidneys.

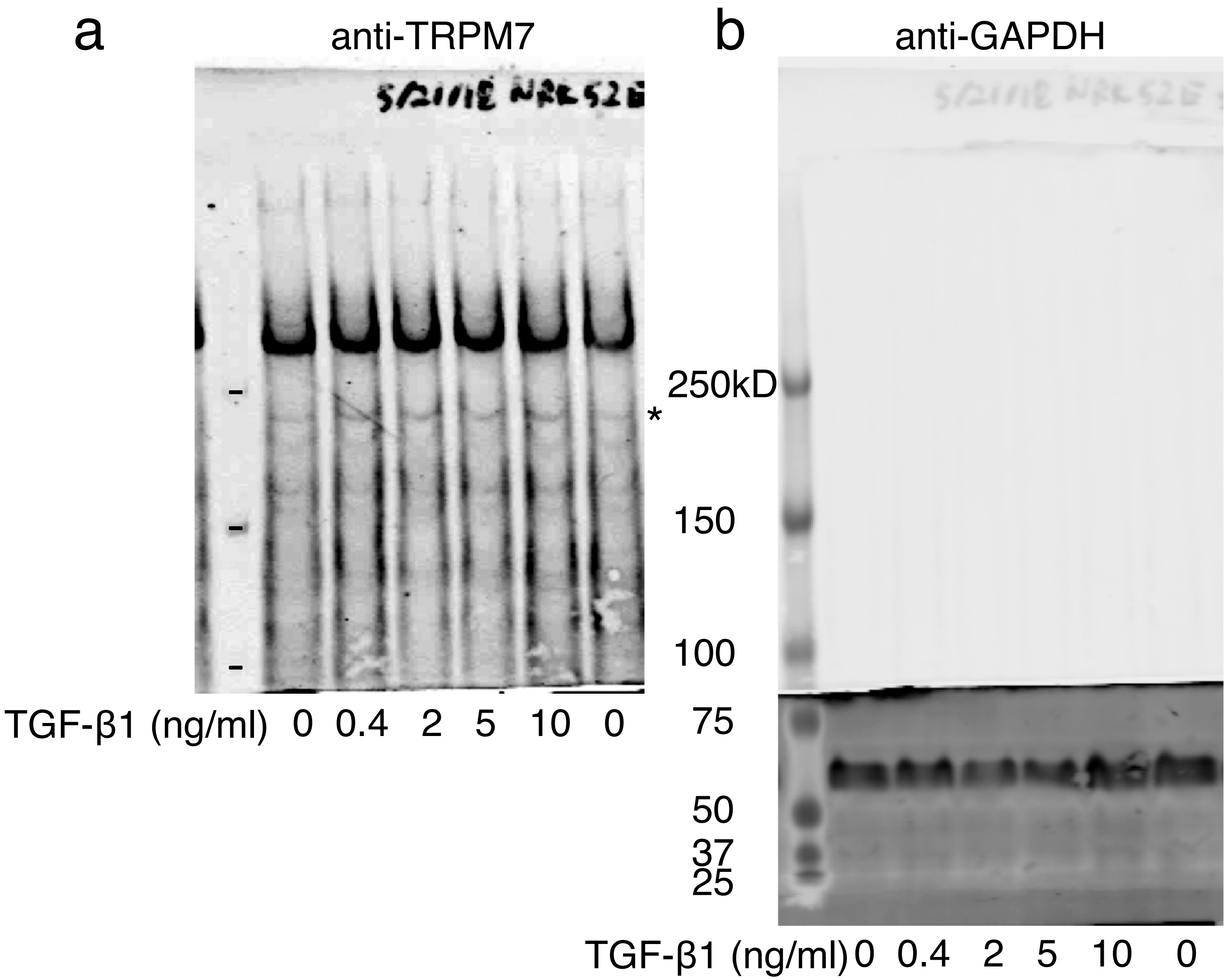

Supplementary Figure S9: TRPM7 protein increased with TGF-β1 treatment in NRK-52E cells.

The full-length western blot of **(a)** TRPM7 and **(b)** GAPDH in Fig. 8h. Asterisk marks the TRPM7 band in (a).
